# Antiplatelet vs. Anticoagulation in Cervical Artery Dissection: A Systematic Review and Meta-Analysis of Randomized Controlled Trials

**DOI:** 10.3389/fneur.2021.745106

**Published:** 2021-11-24

**Authors:** Sihua Liu, Xiao Zhang, Xuesong Bai, Yutong Yang, Tao Wang, Xin Xu, Ran Xu, Long Li, Yao Feng, Kun Yang, Xue Wang, Xiaofan Guo, Jing Chen, Yan Ma, Liqun Jiao

**Affiliations:** ^1^Department of Neurosurgery, Xuanwu Hospital, Capital Medical University, Beijing, China; ^2^China International Neuroscience Institute (China-INI), Beijing, China; ^3^Chinese Academy of Medical Sciences, Peking Union Medical College, Beijing, China; ^4^Imperial College London, National Heart & Lung Institute, London, United Kingdom; ^5^Department of Evidence-Based Medicine, Xuanwu Hospital, Capital Medical University, Beijing, China; ^6^Medical Library, Xuanwu Hospital, Capital Medical University, Beijing, China; ^7^Department of Neurology, Loma Linda University Health, Loma Linda, CA, United States; ^8^Department of Neurology, Zhumadian Central Hospital, Henan, China; ^9^Department of Interventional Neuroradiology, Xuanwu Hospital, Capital Medical University, Beijing, China

**Keywords:** cervical artery dissection, antiplatelet, anticoagulation, ischemic stroke, meta-analysis

## Abstract

**Objective:** The optimal management for cervical artery dissection (CAD) is uncertain. This study aimed to summarize the current randomized controlled trials (RCTs) to compare the efficacy and safety of antiplatelet and anticoagulation therapies for CAD.

**Methods:** A literature search was conducted in the major databases, such as MEDLINE, Embase, and the Cochrane Library. Only the RCTs comparing the antiplatelet and anticoagulation therapies for the patients with CAD were included. Combined estimates of the relative risk (RR) of antiplatelet vs. anticoagulation were analyzed. Heterogeneity was measured using the *I*^2^ statistical analysis. The analyses were performed in the intention-to-treat (ITT) and per-protocol (PP) population, respectively.

**Results:** Two RCTs involving 444 patients in the ITT population and 370 patients in the PP population were included. The quality of studies was high overall. In the ITT population, compared with the patients in the anticoagulation group, the patients in the antiplatelet group showed a higher rate of ischemic stroke within 3 months (*RR* = 6.73 [95% *CI*, 1.22–37.15], *I*^2^ = 0%, *P* = 0.029). No difference between these two treatment groups was found for the outcomes of transient ischemic attack (*RR* = 0.37 [95% *CI*, 0.09–1.58], *I*^2^ = 0%, *P* = 0.181), intracranial hemorrhage (*RR* = 0.33 [95% *CI*, 0.01–7.98], *I*^2^ = 0%, *P* = 0.494), major extracranial bleeding (*RR* = 0.31 [95% *CI*, 0.01–7.60], *I*^2^ = 0%, *P* = 0.476), or the composite of these outcomes within 3 months. For the PP population, the results of the meta-analysis of outcomes between the antiplatelet and anticoagulation groups were consistent with the ITT population.

**Conclusions:** Compared with the antiplatelet group, the anticoagulation group has a lower risk of ischemic stroke without increasing bleeding risk when treating CAD. Anticoagulation seems to be superior over the antiplatelet in treating CAD but needs to be further tested by specifying several issues, such as location, initial symptom types, and treatment protocols.

## Introduction

Cervical artery dissection (CAD), one of the leading causes of stroke in young people, has an incidence rate of 2.6–3.0 per 100,000 population (1). It could present with the variable symptoms, such as pain, headache, and partial Horner's syndrome (2). A major concern of CAD is the increased risk of stroke or transient ischemic attack (TIA), and secondary stroke after initial presentation was estimated to be 15%−20% (3–5). It has been reported that thrombus formation at the dissection site could be a major source of recurrent stroke (6), which usually happened within the first few days after initial symptoms (4, 5). Thus, many clinicians advocate the use of anticoagulation from presentation until 3 or 6 months after the dissection to reduce the risk of early recurrence of stroke (2).

A previous UK questionnaire of diagnosis and treatment in the CAD showed marked variability in decision-making of the clinicians between anticoagulation and antiplatelet (7). Although being hotly debated for the past decade, the optimal management of CAD between the two medication strategies still remains uncertain. The previous meta-analyses showed inconsistent results, mainly due to the unavoidable biases from solely recruiting non-randomized controlled trials (non-RCTs) (8–12). The American Heart Association/American Stroke Association (AHA/ASA) 2019 guidelines open for both antiplatelet and anticoagulation were based on the low evidence level (IIa, B-NR) (13). Recently, two RCTs, antiplatelet therapy vs. anticoagulation therapy in CAD-The Cervical Artery Dissection in Stroke Study (CADISS) (14) and aspirin vs. anticoagulation in CAD (TREAT-CAD) (15), showed different results. CADISS found no difference in efficacy of anticoagulation and antiplatelet at preventing stroke and death in the symptomatic patients with CAD (14), but TREAT-CAD failed to show non-inferiority of antiplatelet using aspirin compared with anticoagulation in the treatment of CAD (15). Thus, an updated systematic review and meta-analysis by recruiting only RCTs is necessary to provide the clinicians with more reliable evidence of treating CAD. This systematic review and meta-analysis summarized the current literature to provide the most up-to-date and reliable clinical evidence of anticoagulation and antiplatelet therapies in the patients with CAD based on RCTs.

## Methods

This study was reported following the Preferred Reporting Items for Systematic Reviews and Meta-Analyses (PRISMA) guidelines (16). The ethics approval and informed consent were not required due to the study design.

### Literature Search Strategy

A comprehensive literature search of MEDLINE, the Cochrane Library, Embase, and clinical trial registers (such as, ClinicalTrials.gov) was conducted by the two independent reviewers (SL and XX). The following keywords were used: “carotid artery injuries,” “carotid artery, internal, dissection,” “vertebral artery dissection,” “aneurysm,” “pseudoaneurysm,” “dissection,” “antiplatelet,” and “anticoagulant.” There were no restrictions regarding the language or publication status. The last literature search was performed on May 28, 2021. A detailed search strategy was shown in Supplementary Appendix 1.

### Study Selection

The population, intervention, comparator, outcome, and study design (PICOS) model was utilized to determine the criteria of studies included. Two reviewers (SL and XX) independently selected the eligible studies by screening the titles/abstracts first and then reading the full articles. A third reviewer (TW) resolved the disagreement between the two reviewers.

#### Population

##### Inclusion Criteria

The patients aged 18 years or older. The patients had extracranial carotid or vertebral artery dissection with the onset of symptom within 2 weeks, and imaging evidence of definite or probable dissection. Imaging evidence had to be confirmed by magnetic resonance imaging (MRI) or angiography (MRA), computed tomography angiography (CTA), or intra-arterial angiography.

##### Exclusion Criteria

The patients with intracranial CAD, contraindications to use of the antiplatelet or anticoagulation drugs, and pregnancy.

#### Intervention

The interventions should include the antiplatelet and anticoagulation therapies for at least 3 months. In the antiplatelet group, the patients received aspirin, clopidogrel, or dipyridamole, or in dual combination. In the anticoagulation group, the patients received warfarin, acenocoumarol, or phenprocoumon with a target international normalized ratio (INR) of 2.0–3.0. Heparin or low-molecular-weight heparin (LMWH) can be used as a bridging treatment until the target INR had been reached.

#### Comparator

The comparator was antiplatelet therapy vs. anticoagulation therapy.

#### Outcome

At least one of the following items was reported:

1. Ischemic stroke within 3 months
2. Transient ischemic attack within 3 months
3. Intracranial hemorrhage (ICH) within 3 months
4. Major extracranial bleeding within 3 months
5. Any cause of death within 3 months
6. Composite of the above clinical outcomes

#### Study Design

Only RCTs were included. Other study types, such as the case-control studies or cohort studies, reviews, abstracts, conference reports, and case reports were excluded.

### Data Extraction

Data concerning the trial design, baseline characteristics, and outcomes of each included study were independently extracted by the two reviewers (RX and LL). A third reviewer (TW) was invited for the resolution of discrepancies between the two reviewers. We attempted to contact the corresponding authors of the study if there were missing or ambiguous data.

### Assessment of Risk of Bias and Heterogeneity

Two independent reviewers (XW and KY) evaluated the risk of bias in the included studies using the Cochrane Collaboration criteria (RoB tool V.2) (17). Heterogeneity was measured using the *I*^2^ statistical analysis. Substantial heterogeneity was defined as an *I*^2^ statistic of >60%. If the *I*^2^ statistic was ≥20%, the DerSimonian and Laird method of the random-effects model was applied for pooling outcomes; otherwise, the Mantel–Haenszel method of the fixed-effects model was performed (18).

### Statistical Analysis

A meta-analysis of the outcomes was conducted only when there was a sufficient sample size in the two or more studies. The analyses were performed in the intention-to-treat (ITT) and per-protocol (PP) population, respectively. The PP population excluded the patients who did not meet the inclusion criteria and consisted of the patients who received the allocated treatment and completed the assessment period. The results were reported as relative risk (RR) with a 95% *CI* for categorical data. The forest plots were used for the graphical representations. The sensitivity analyses were used to explore the heterogeneity of the outcomes. The funnel plots were used to evaluate the publication bias of the included studies. The differences were considered statistically significant at an overall *P* < 0.05. Stata software (version 15.0, Stata Corp, College Station, TX, USA) was used for all the data analyses.

## Results

### Study Selection and Characteristics

**Figure 1:**
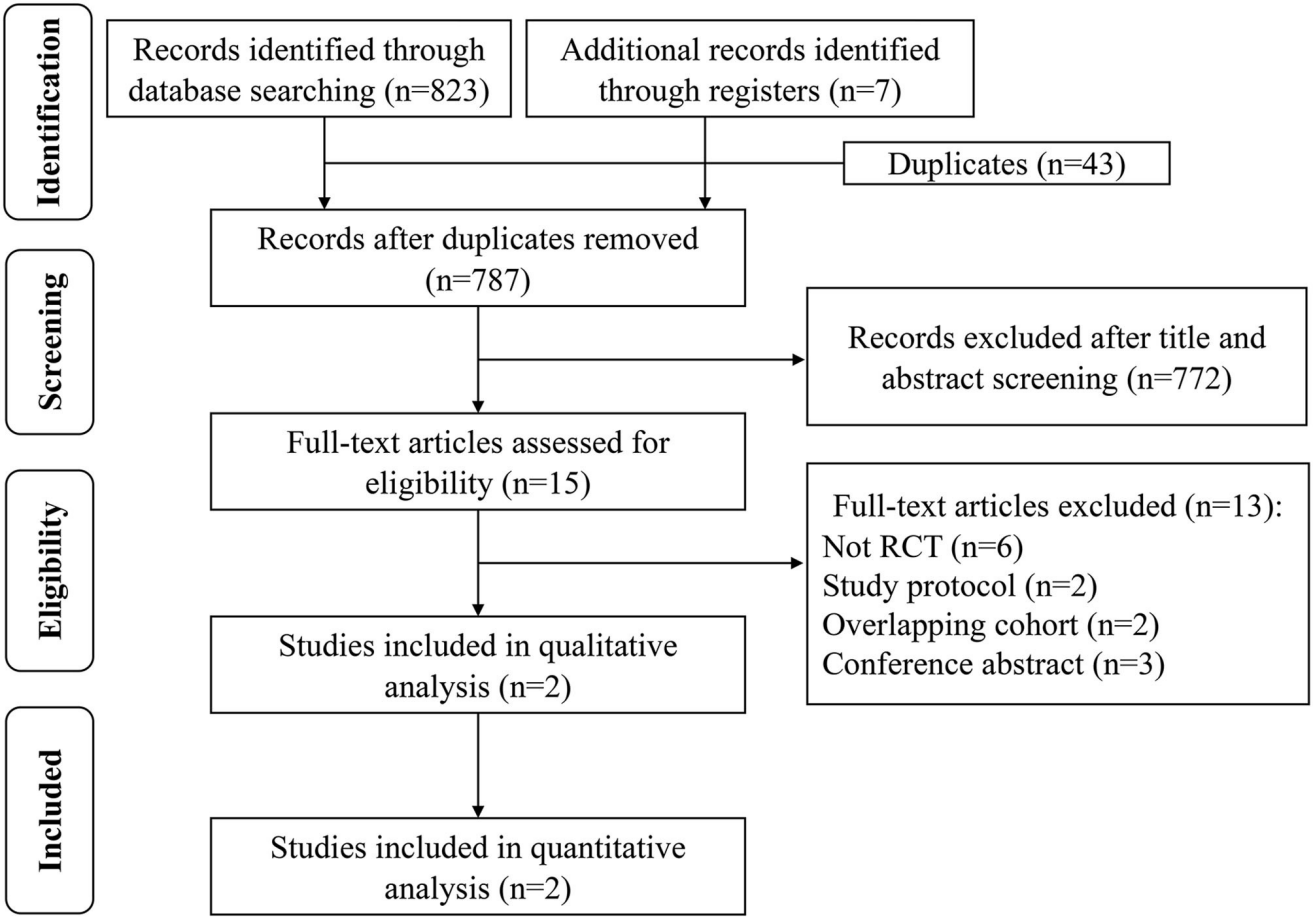
Flow diagram of literature for the meta-analysis.

A total of 830 potential studies were initially identified, of which only two RCTs were eligible for the qualitative and quantitative analysis following the reviewer evaluations. The two studies consisted of CADISS (14) and TREAT-CAD (15). A flow diagram of the search procedure is shown in Figure 1.

**Figure 2:**
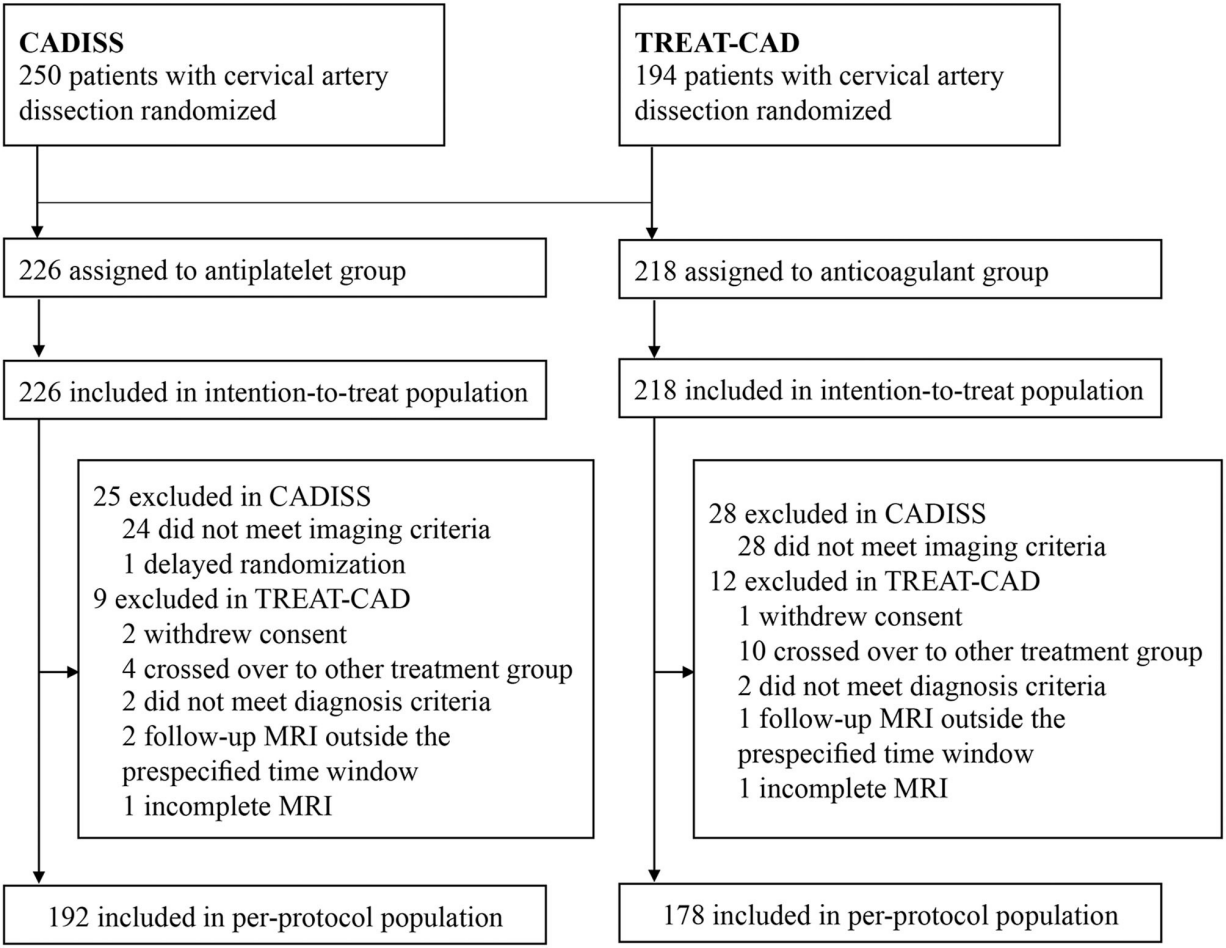
Study profile.

From these two trials, a total of 444 patients with CAD were included in the ITT population and 370 patients were included in the PP population (Figure 2). In CADISS, the patients with extracranial carotid and vertebral dissection within 7 days of onset of symptoms were randomized to receive the antiplatelet or anticoagulant treatment for 3 months; in TREAT-CAD, the patients who had symptomatic CAD within 14 days were randomized to receive either aspirin or a vitamin K antagonist for 3 months. In CADISS, 52 patients were excluded in the PP population because they did not meet the imaging criteria after randomization (24 in the antiplatelet group and 28 in the anticoagulation group), and one patient in the antiplatelet group was excluded in the PP population because of delayed randomization; in TREAT-CAD, 21 patients were not included in the PP population (9 in the antiplatelet group and 12 in the anticoagulation group). Therefore, the PP population included 370 patients (192 in the antiplatelet group and 178 in the anticoagulant group).

The characteristics of the included trials and the patients are summarized in Table 1. Both these trials were multicenter published after 2018. The recruitment period was between 2006 and 2018. They were conducted in Europe and Oceania. Among the ITT population, 248 (56%) patients had carotid artery dissection and 199 (45%) patients had vertebral artery dissection, including 3 (1%) patients with the carotid artery plus the vertebral artery dissection. The mean age of the patients ranged from 45.5 to 49.3 years. The most presenting local sign and symptom was headache in 303 (68%) patients and 295 (66%) of 444 patients presented with ischemic stroke. The mean time between the symptoms and randomization was 3.65 days in CADISS, and the median time between the symptoms and treatment was 7 days in TREAT-CAD. The mean modified Rankin score was between 1.8 and 2.2. In CADISS, 55 (44%) of 126 patients in the antiplatelet group received aspirin combined with clopidogrel or dipyridamole, 42 (33%) received clopidogrel alone, 28 (22%) received aspirin alone, one (1%) received dipyridamole alone; 112 (90%) of 124 patients in the anticoagulant group received heparin or LMWH followed by warfarin, and 12 (10%) received warfarin only. In TREAT-CAD, all the patients received aspirin 300 mg once daily alone in the antiplatelet group; 51 (54%) of 94 patients in the anticoagulant group received heparin or LMWH followed by a vitamin K antagonist and 43 (46%) received a vitamin K antagonist only. In addition, 8.8 and 13.9% of the patients received acute recanalization therapy in the CADISS and TREAT-CAD trials, respectively. The follow-up of CADISS and TREAT-CAD trials was 12 and 3 months, respectively.

**Table 1:** The characteristics of the included studies and patients.

**Population**	**ITT population**				**PP population**			
Study	CADISS (14)		TREAT-CAD (15)		CADISS (14)		TREAT-CAD (15)	
Publication time	2019		2021		2019		2021	
Recruitment period	2006–2013		2013–2018		2006–2013		2013–2018	
Follow-up, months	12		3		12		3	
Country	United Kingdom and Australia		Switzerland, Germany, and Denmark		United Kingdom and Australia		Switzerland, Germany, and Denmark	
Number of included patients	Antiplatelets	Anticoagulants	Antiplatelets	Anticoagulants	Antiplatelets	Anticoagulants	Antiplatelets	Anticoagulants
	(*n* = 126)	(*n* = 124)	(*n* = 100)	(*n* = 94)	(*n* = 101)	(*n* = 96)	(*n* = 91)	(*n* = 82)
Number of crossed over to other treatment group, *n* (%)	0 (0)	0 (0)	4 (4)	10 (11)	0 (0)	0 (0)	0 (0)	0 (0)
Age, mean (SD), year	49.3 (12)	49.2 (12)	46.6 (10.6)	45.5 (11.6)	48.5 (12)	48.1 (11)	46.7 (10.2)	45.5 (11.6)
Male, n (%)	87 (69)	87 (70)	62 (62)	61 (65)	69 (68)	66 (69)	56 (62)	54 (66)
**Site of dissection**, ***n*** **(%)**								
Internal carotid	58 (46)	60 (48)	72 (72)	58 (62)	51 (51)	47 (49)	65 (71)	50 (61)
Vertebral	68 (54)	64 (52)	29 (29)	38 (40)	50 (50)	49 (51)	27 (30)	34 (41)
**Presenting signs and symptoms**, ***n*** **(%)**								
Amaurosis fugax	4 (3)	5 (4)	2 (2)	7 (7)	4 (4)	4 (4)	2 (2)	5 (6)
Retinal infarction	0 (0)	1 (0.8)	3 (3)	1 (1)	0 (0)	1 (1)	3 (3)	1 (1)
TIA	27 (21)	20 (16)	14 (14)	10 (11)	20 (20)	15 (16)	12 (13)	10 (12)
Ischemic stroke	93 (74)	101 (82)	52 (52)	49 (52)	74 (73)	77 (80)	47 (52)	43 (52)
Headache	84 (67)	83 (67)	72 (72)	64 (68)	68 (67)	68 (71)	65 (71)	54 (66)
Neck pain	57 (45)	63 (51)	51 (51)	47 (50)	41 (41)	51 (53)	46 (51)	41 (50)
Horner syndrome	26 (20.6)	34 (27.4)	36 (36)	34 (36)	24 (24)	29 (30)	32 (35)	28 (34)
Time between symptoms and randomization/treatment, mean (SD), or median (IQR), day	3.9 (1.8)	3.4 (2.0)	7.0 (4.0–10.0)	6.0 (4.0–9.0)	3.8 (1.8)	3.3 (2.1)	7.0 (4.0–10.0)	6.0 (3.2–8.8)
Modified Rankin score, mean (SD)	2.1 (1.5)	2.1 (1.5)	1.8 (1.2)	1.8 (1.3)	2.1 (1.6)	2.2 (1.5)	1.8 (1.2)	1.8 (1.3)
Acute recanalization therapy, n (%)	12 (10)	10 (8)	16 (16)	11 (12)	10 (10)	8 (8)	15 (16)	8 (10)
**Risk factors, n (%)**								
Hypertension	29 (23)	26 (21)	32 (32)	28 (30)	21 (wq)	19 (20)	30 (33)	25 (30)
Diabetes mellitus	5 (4)	5 (4)	1 (1)	3 (3)	3 (3)	3 (3)	1 (1)	3 (4)
Hypercholesterolaemia	16 (13)	19 (15)	19 (19)	20 (21)	12 (12)	11 (12)	18 (20)	18 (22)
Smoking history	63 (50)	66 (53)	46 (46)	47 (50)	52 (52)	51 (53)	43 (47)	42 (51)
Migraine	20 (16)	25 (20)	31 (31)	19 (20)	15 (15)	22 (23)	30 (33)	17 (21)
Mechanical trigger event within 4 weeks before enrolment	32 (25)	21 (17)	13 (13)	18 (19)	26 (26)	16 (17)	12 (13)	16 (20)

*ITT, intention-to-treat; PP, per-protocol, SD, standard deviation; TIA, transient ischemic attack; IQR, interquartile range*.

### The Meta-Analysis of the Outcomes of Antiplatelet and Anticoagulation

The meta-analysis of the outcomes within 3 months of the antiplatelet and anticoagulation groups in the ITT and PP population is summarized in Table 2. In the ITT population, the pooled proportion of ischemic stroke was 4.15% (95% *CI*, 1.82%−7.26%) in the antiplatelet and 0.31% (95% *CI*, 0%−1.81%) in the anticoagulation groups; the pooled proportion of TIA was 0.61% (95% *CI*, 0%−2.30%) in antiplatelet and 2.72% (95% *CI*, 0.84%−5.46%) in the anticoagulation groups; the estimated incidences of ICH were 0% (95% *CI*, 0%−0.85%) in antiplatelet and 0.31% (95% *CI*, 0%−1.81%) in the anticoagulation groups; the estimated incidences of major extracranial bleeding were 0% (95% *CI*, 0%−0.85%) in antiplatelet and 0.25% (95% *CI*, 0%−1.67%) in the anticoagulation groups; the composite of ischemic stroke, ICH, or death was 4.15% (95% *CI*, 1.82%−7.26%) in antiplatelet and 0.64% (95% *CI*, 0%−2.4%) in the anticoagulation groups. There were no deaths within 3 months in both the antiplatelet and anticoagulation groups.

**Table 2:** Summary of the meta-analysis of the outcomes within 3 months of the antiplatelet and anticoagulation therapies.

	**ITT population**		**PP population**	
**Outcomes**	**Antiplatelets (%) (95% CI)**	**Anticoagulations (%) (95%CI)**	**Antiplatelets (%) (95% CI)**	**Anticoagulations (%) (95%CI)**
Ischemic stroke	4.15 (1.82–7.26)	0.31 (0–1.81)	4.96 (2.21–8.62)	0.37 (0–2.19)
TIA	0.61 (0–2.30)	2.72 (0.84–5.46)	0.69 (0–2.65)	2.8 (0.71–5.93)
ICH	0 (0–0.85)	0.31 (0–1.81)	0 (0–1)	0.37 (0–2.19)
Major extracranial bleeding	0 (0–0.85)	0.25 (0–1.67)	0 (0–1)	0.33 (0–2.09)
Death	0 (0–0.85)	0 (0–0.88)	0 (0–1)	0 (0–1.08)
Ischemic stroke, ICH, or death	4.15 (1.82–7.26)	0.64 (0–2.4)	4.96 (2.21–8.62)	0.76 (0–2.88)
Ischemic stroke or ICH	4.15 (1.82–7.26)	0.64 (0–2.4)	4.96 (2.21–8.62)	0.76 (0–2.88)
Ischemic stroke or TIA	5.21 (2.58–8.59)	3.15 (1.11–6.02)	6.18 (3.09–10.15)	3.32 (1.02–6.65)
Ischemic stroke, ICH, or TIA	5.21(2.58–8.59)	3.56 (1.38–6.56)	6.18 (3.09–10.15)	3.83 (1.34–7.32)

*ITT, intention-to-treat; PP, per-protocol; TIA, transient ischemic attack; ICH, intracranial hemorrhage; CI, confidence interval*.

### Comparison of the Outcomes Between Antiplatelet and Anticoagulation

**Figure 3:**
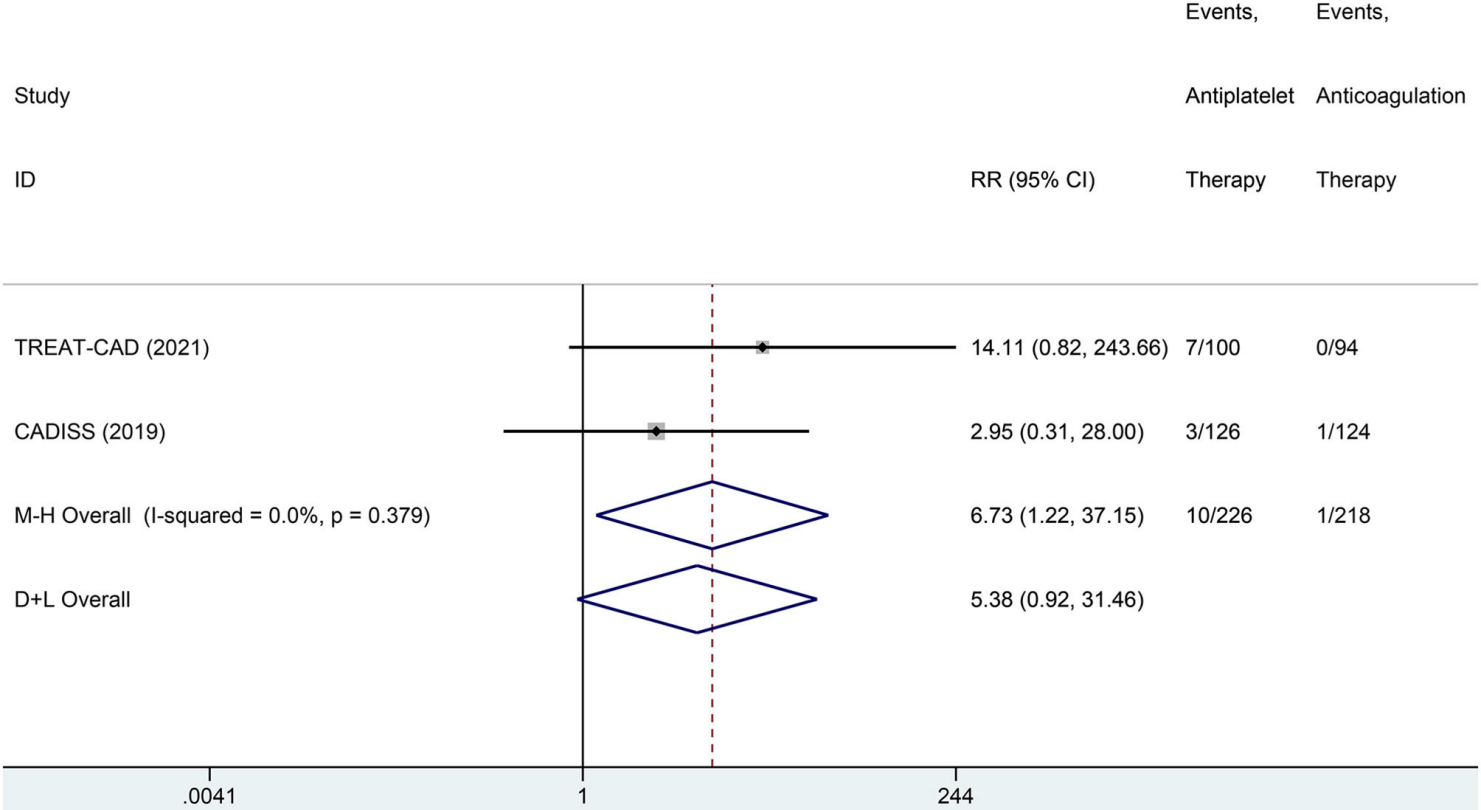
A forest plot of comparison for ischemic stroke between the antiplatelet and anticoagulation in the intention-to-treat (ITT) population.

**Figure 4:**
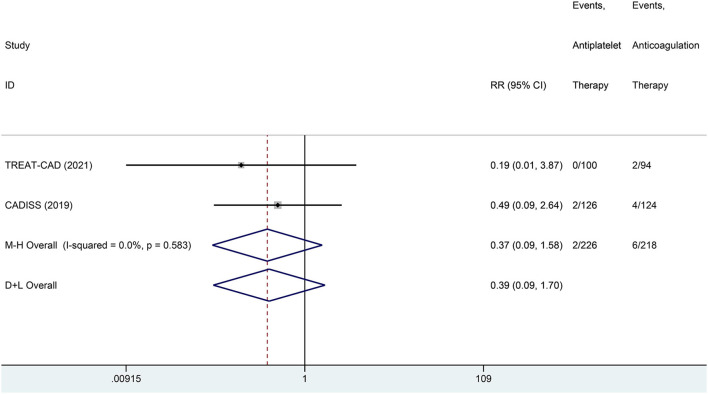
A forest plot of comparison for transient ischemic attack (TIA) between the antiplatelet and anticoagulation in the ITT population.

**Figure 5:**
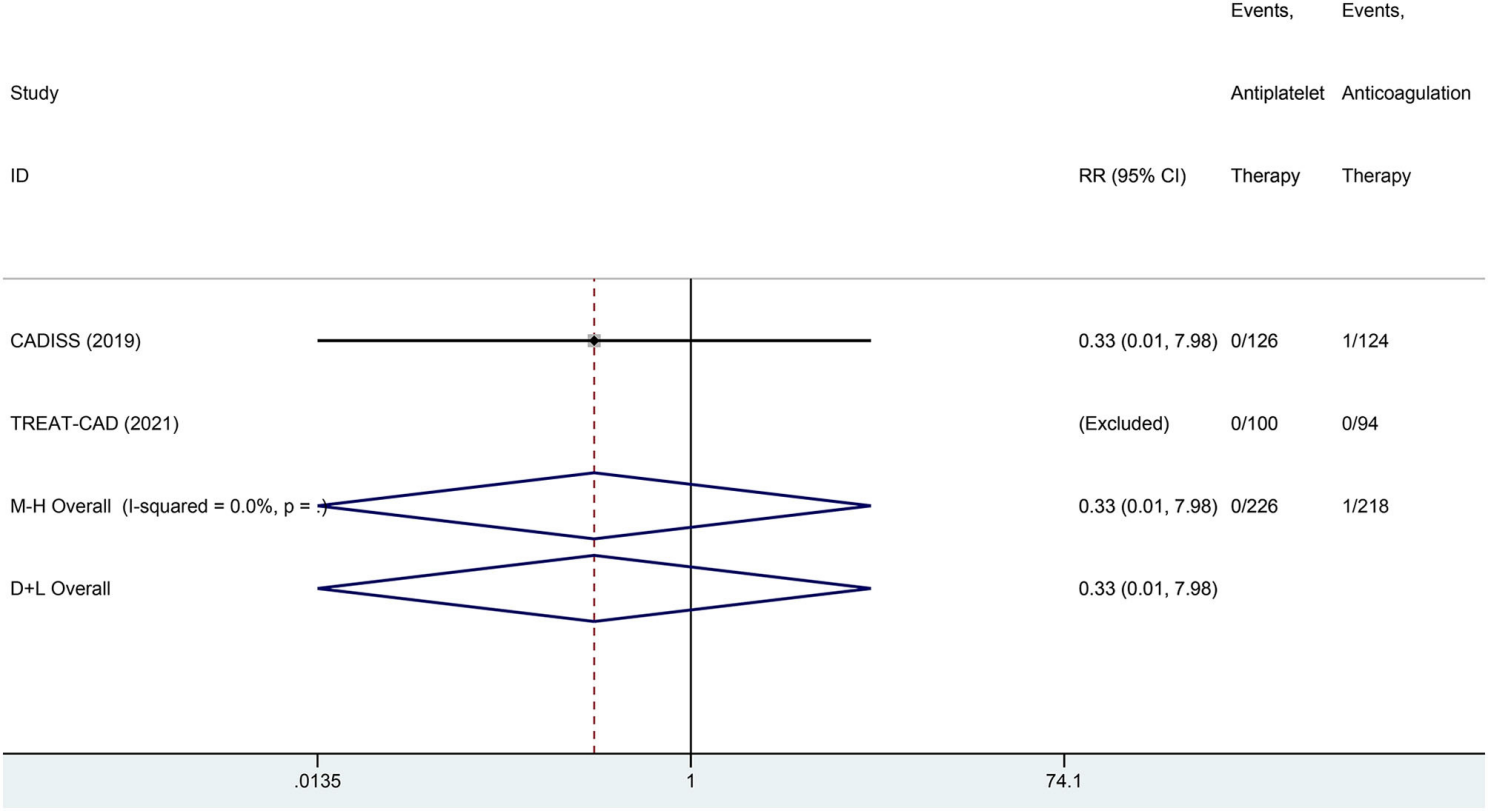
A forest plot of comparison for intracranial hemorrhage (ICH) between the antiplatelet and anticoagulation in the ITT population.

**Figure 6:**
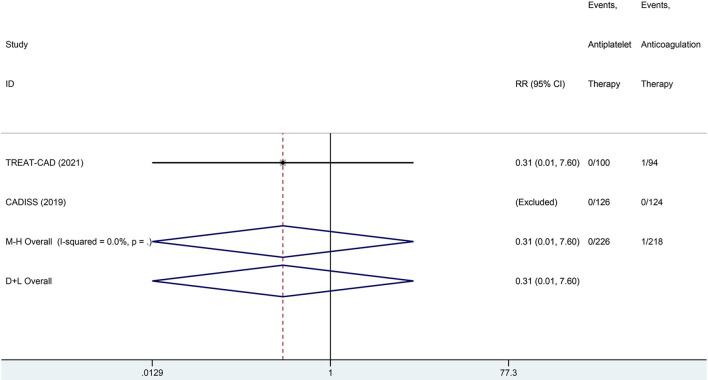
A forest plot of comparison for major extracranial bleeding between the antiplatelet and anticoagulation in the ITT population.

We conducted a meta-analysis comparing the outcomes of antiplatelet and anticoagulation in the ITT population (Table 3). The rate of ischemic stroke within 3 months in the antiplatelet group was higher than that in the anticoagulation group (*RR* = 6.73 [95% *CI*, 1.22–37.15], *I*^2^ = 0%, *P* = 0.029; Figure 3). No difference between the treatment groups was found for the outcomes of TIA (*RR* = 0.37 [95% *CI*, 0.09–1.58], *I*^2^ = 0%, *P* = 0.181; Figure 4), ICH (*RR* = 0.33 [95% *CI*, 0.01–7.98], *I*^2^ = 0%, *P* = 0.494; Figure 5), major extracranial bleeding (*RR* = 0.31 [95% *CI*, 0.01–7.60], *I*^2^ = 0%, *P* = 0.476; Figure 6), or the composite of these outcomes within 3 months (Supplementary Figures 1–4). The results of the meta-analysis of comparative outcomes within 3 months between the antiplatelet and anticoagulation conducted in the PP population were consistent with the ITT population (Supplementary Table 1).

**Table 3:** The outcomes of comparison between the antiplatelet and anticoagulation therapies in intention-to-treat (ITT) population.

**Outcomes**	**Pooled RR**	**95% CI**	***I** ^2^* **(%)**	***P*** **value**
Ischemic stroke	6.73	1.22–37.15	0	0.029
TIA	0.37	0.09–1.58	0	0.181
ICH	0.33	0.01–7.98	0	0.494
Major extracranial bleeding	0.31	0.01–7.60	0	0.476
Death^*^				
Ischemic stroke, ICH or death	3.55	0.35–35.49	49.6	0.281
Ischemic stroke or ICH	3.55	0.35–35.49	49.6	0.281
Ischemic stroke or TIA	1.63	0.51–5.27	31.6	0.413
Ischemic stroke, ICH or TIA	1.49	0.38–5.78	50.1	0.563

*ITT, intention-to-treat; TIA, transient ischemic attack; ICH, intracranial hemorrhage; RR, risk ratio; CI, confidence interval*.

*I^2^ the variation attributable to heterogeneity*.

* *This outcome was excluded because data were unable to analyze*.

### Risk of Bias

Through Risk of Bias (RoB) tool V.2 assessment, the CADISS trial showed a low risk of bias in all the five domains, but the TREAT-CAD showed a low risk of bias in the four domains and a moderate risk of bias in the domain of deviations from the intended interventions because there were some patients crossed over between the treatment groups (Supplementary Figure 5). None of the substantial heterogeneity was observed in all these comparative outcomes. The sensitivity analyses and funnel plots of the outcomes are presented in the Supplementary Appendix 3.

## Discussion

This is the first systematic review and meta-analysis comparing the anticoagulation and antiplatelet in CAD based on the RCTs. Both the analyses in ITT and PP population showed consistent results. The final results showed that the anticoagulation is significantly more effective in preventing ischemic stroke than antiplatelet (ITT, 0.31% vs. 4.15%). All the cases of major bleeding occurred in the anticoagulation group, such as ICH (0.31%) and major extracranial bleeding (0.25%), but no difference was found between both the groups. Although the anticoagulation group had a numerically higher risk of TIA (ITT, 2.72 vs. 0.61%), there was no significant difference between the two groups. Additionally, the composite outcome of ischemic stroke, ICH, or death was numerically higher in the antiplatelet group rather than the anticoagulation group (ITT, 4.15 vs. 0.64%) without significant difference.

Ischemic stroke is a major concern of CAD and has been reported as mostly occurring within the first few weeks after initial symptoms. In this meta-analysis, both the groups had a low risk of ischemic stroke, with anticoagulation being more effective in its prevention. In the TREAT-CAD trial, all the seven cases of ischemic strokes occurred in the antiplatelet group. Furthermore, in the CADISS trial, ischemic stroke occurred numerically more often in the antiplatelet group than in the anticoagulation group (2 vs. 1%). The researchers from the TREAT-CAD trial proposed recommended bridging with heparin or LMWH in addition to a vitamin K antagonist in the patients of the anticoagulation group before reaching the target INR might have had better protection against early ischemic stroke than the patients in the antiplatelet group. Another reason may be the variation in the dosage of antiplatelet medications. In the TREAT-CAD trial, all the patients received aspirin as monotherapy in a higher daily dose of 300 mg. While in the CADISS trial, 44% of the patients in the antiplatelet group received aspirin plus clopidogrel or dipyridamole. Additionally, we found a numerically higher rate of TIA in the anticoagulation group. A retrospective analysis comparing aspirin and anticoagulation in carotid artery dissection reported a similar result: 8 (4.0%) of 202 patients in the anticoagulation group and 2 (2.1%) of 96 patients in the antiplatelet group occurred TIA (19). The reasons remain uncertain. One possible explanation may be that the increased number of TIA indirectly proves higher potency of anticoagulation by controlling the ischemic events to a less severe degree. However, the previous meta-analyses showed different results from the current study. In the meta-analysis of Chowdhury et al. (8), the ischemic stroke rate was comparable between the anticoagulation group and antiplatelet group (1.74 vs. 1.43%). This was further supported by another meta-analysis (antiplatelet 2.6% and anticoagulation 1.8%) (10). Consequently, the guidelines remained open for both the treatments based on low-level clinical evidence (13). So, the unexpected results from the current meta-analysis may require the clinicians to revisit this question in the future.

Another issue of concern is bleeding risk and the risk-benefit analysis between the two treatments. In the current study, no difference was found between the two groups regarding ICH or extracranial hemorrhage. In addition, there was no difference between the two groups regarding the composite outcome of ischemic stroke, ICH, or TIA, although it was numerically higher in the antiplatelet group (5.21 vs. 3.56%). All the severe bleeding cases occurred in the anticoagulation group. In the TREAT-CAD trial, one patient (1%) in the anticoagulation group had a major extracranial hemorrhage. In the CADISS trial, one major bleeding (1%) was subarachnoid hemorrhage in the anticoagulation group. But the general risk of bleeding may be acceptable considering its low frequency and its similarity to the previous meta-analysis of Chowdhury et al. (8). In that study, either symptomatic ICH or major extracranial hemorrhages only occurred in the anticoagulation group (symptomatic ICH, 0.72% (5/697); major extracranial hemorrhages, 1.42% (7/495); respectively, and no difference was found between the two treatments (8). Also, the death and disability comparison showed no benefit of antiplatelet therapy (8). Therefore, the safety of anticoagulation may seem not to be a concern. While, in another meta-analysis of Sarikaya et al. (9), the primary composite outcome of ischemic stroke, ICH, or death within the first 3 months after treatment initiation favors the antiplatelets. The observed difference may be due to bias from the observational studies. However, as these observational studies may reflect the real-world settings, we should still draw the conclusions with caution.

There are several limitations of the current meta-analysis. First, a tenth of the ITT population was excluded in the PP population mainly due to the unconfirmed diagnosis of CAD and the cross-over treatment, which might cause the potential biases, while all these results in the ITT and PP populations were consistent. Furthermore, the location of CAD, extracranial carotid, and vertebral artery could not be discriminated when comparing antiplatelet with anticoagulation. Additionally, the patients with initial symptoms of stroke could not be analyzed separately, as initial symptom type influences the chance of recurrent ischemic events (19, 20). Additionally, the different antiplatelet protocols existed between the recruited two RCTs. None used direct oral anticoagulants in the anticoagulation group, which may have a more favorable risk-benefit ratio and are more conveniently applicable than vitamin K antagonists (21, 22). In addition, the CADISS trial has 1-year final results beyond a 3-month period and the TREAT-CAD trial evaluated the MRI outcomes (new ischemic or hemorrhagic brain lesions). These may hold the clinical values and should be further explored in future studies.

## Conclusions

In this meta-analysis, recruiting only the RCTs, anticoagulation has a lower risk of ischemic stroke without increasing the bleeding risk when treating CAD. Antiplatelet has a numerically higher risk of a composite outcome of ischemic stroke, ICH, or death. Anticoagulation seems to be superior over antiplatelet in treating CAD but needs to be further tested by specifying several issues, such as location, initial symptom types, and the treatment protocols.

## Data Availability Statement

The original contributions presented in the study are included in the article/Supplementary Material, further inquiries can be directed to the corresponding authors.

## Author Contributions

XZ, XB, and LJ developed the initial ideas for this study and formulated the study designs. SL, TW, XX, RX, LL, XW, and KY performed the literature search and data analysis. LJ, YM, XG, and JC were consulted about clinical issues. SL, XZ, and XB contributed to the original drafts. SL, XZ, XB, YY, TW, XX, RX, LL, YF, KY, XW, XG, JC, YM, and LJ were responsible for the revision of the draft. All authors read and approved the final version of the manuscript prior to submission.

## Funding

This work was supported by the Beijing Scientific and Technologic Project (Z201100005520019), Beijing, China.

## Conflict of Interest

The authors declare that the research was conducted in the absence of any commercial or financial relationships that could be construed as a potential conflict of interest.

## Publisher's Note

All claims expressed in this article are solely those of the authors and do not necessarily represent those of their affiliated organizations, or those of the publisher, the editors and the reviewers. Any product that may be evaluated in this article, or claim that may be made by its manufacturer, is not guaranteed or endorsed by the publisher.
