# Supplementary material for: Antiplatelet vs. Anticoagulation in Cervical Artery Dissection: A Systematic Review and Meta-Analysis of Randomized Controlled Trials
Source: Front Neurol. 2021 Nov 24;12:745106. doi: 10.3389/fneur.2021.745106 (PMC8651981; doi:10.3389/fneur.2021.745106)

**Supplementary file 2.**

**Supplementary Table 1.** The outcomes of comparison between antiplatelet and anticoagulation therapies in PP population.

| Outcomes | Pooled RR | 95% CI | *I^2^*(%) | *P* value |
| --- | --- | --- | --- | --- |
| Ischemic stroke | 6.47 | 1.17–35.72 | 0 | 0.032 |
| TIA | 0.42 | 0.10–1.86 | 0 | 0.256 |
| ICH | 0.32 | 0.01–7.69 | 0 | 0.480 |
| Major extracranial bleeding | 0.30 | 0.01–7.28 | 0 | 0.460 |
| Death* |  |  |  |  |
| Ischemic stroke, ICH or death | 3.41 | 0.34–33.91 | 49.4 | 0.295 |
| Ischemic stroke or ICH | 3.41 | 0.34–33.91 | 49.4 | 0.295 |
| Ischemic stroke or TIA | 1.85 | 0.71–4.86 | 0 | 0.209 |
| Ischemic stroke, ICH or TIA | 1.57 | 0.49–5.03 | 31.3 | 0.449 |

*PP* per-protocol, *TIA* transient ischemic attack, *ICH* intracranial hemorrhage, *RR* risk ratio, *CI* conﬁdence interval, *I*^2^ the variation attributable to heterogeneity.

*This outcome was excluded because data were unable to analysis.

**Supplementary Figure 1.** Forest plot of comparison for ischemic stroke, ICH, or death within 3 months between antiplatelet and anticoagulation in the ITT population

**
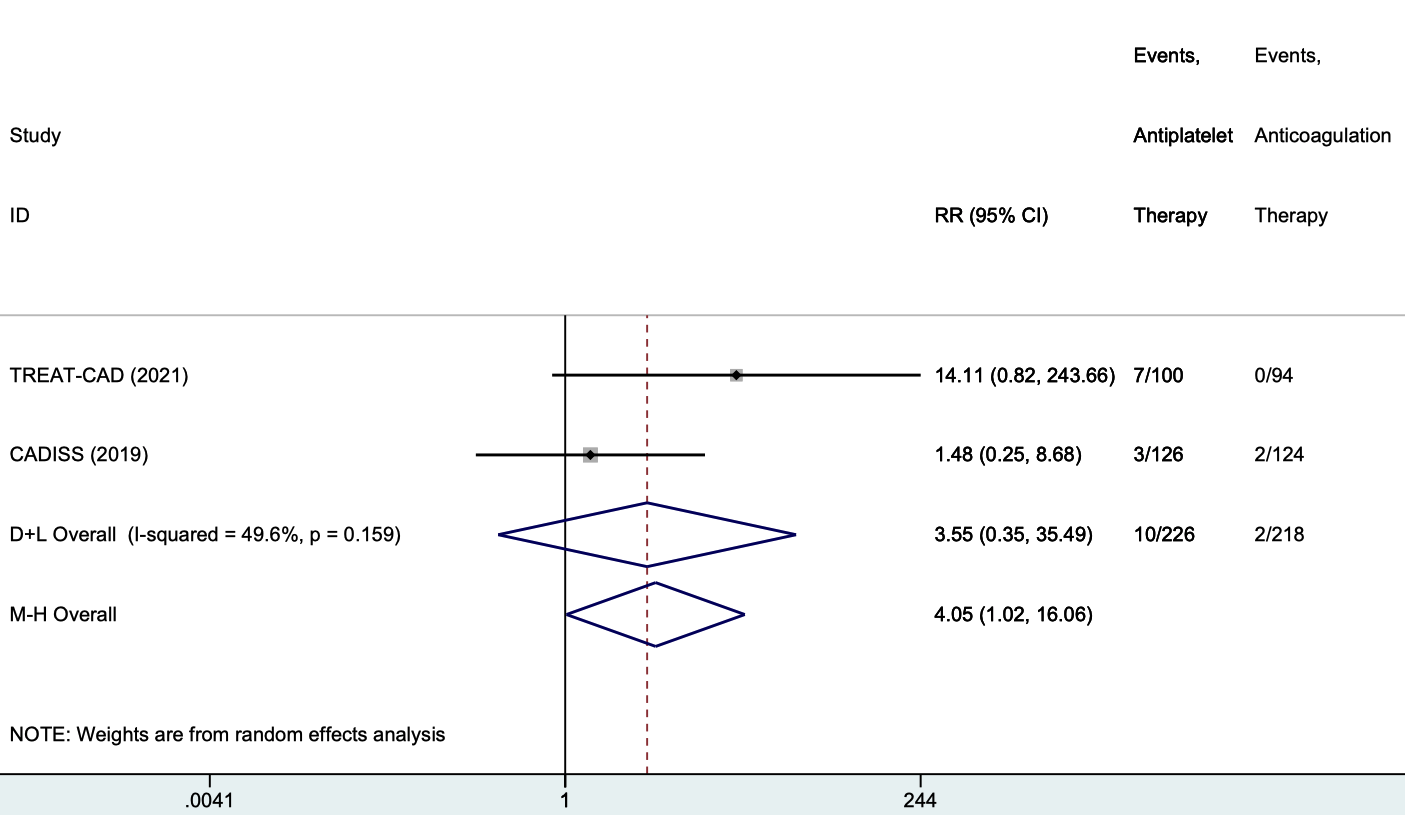
**

**Supplementary Figure 2.** Forest plot of comparison for ischemic stroke or ICH within 3 months between antiplatelet and anticoagulation in the ITT population

**
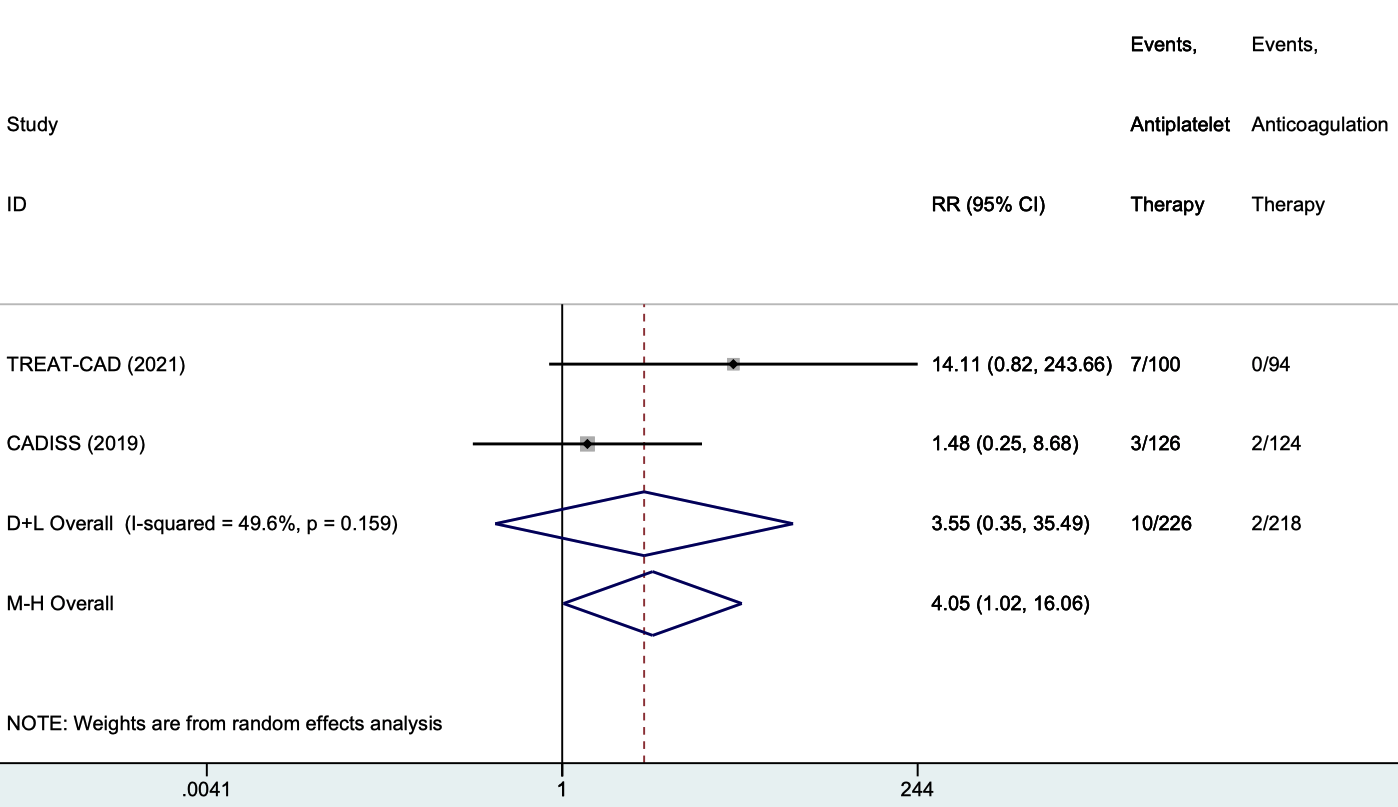
**

**Supplementary Figure 3.** Forest plot of comparison for ischemic stroke or TIA within 3 months between antiplatelet and anticoagulation in the ITT population

**
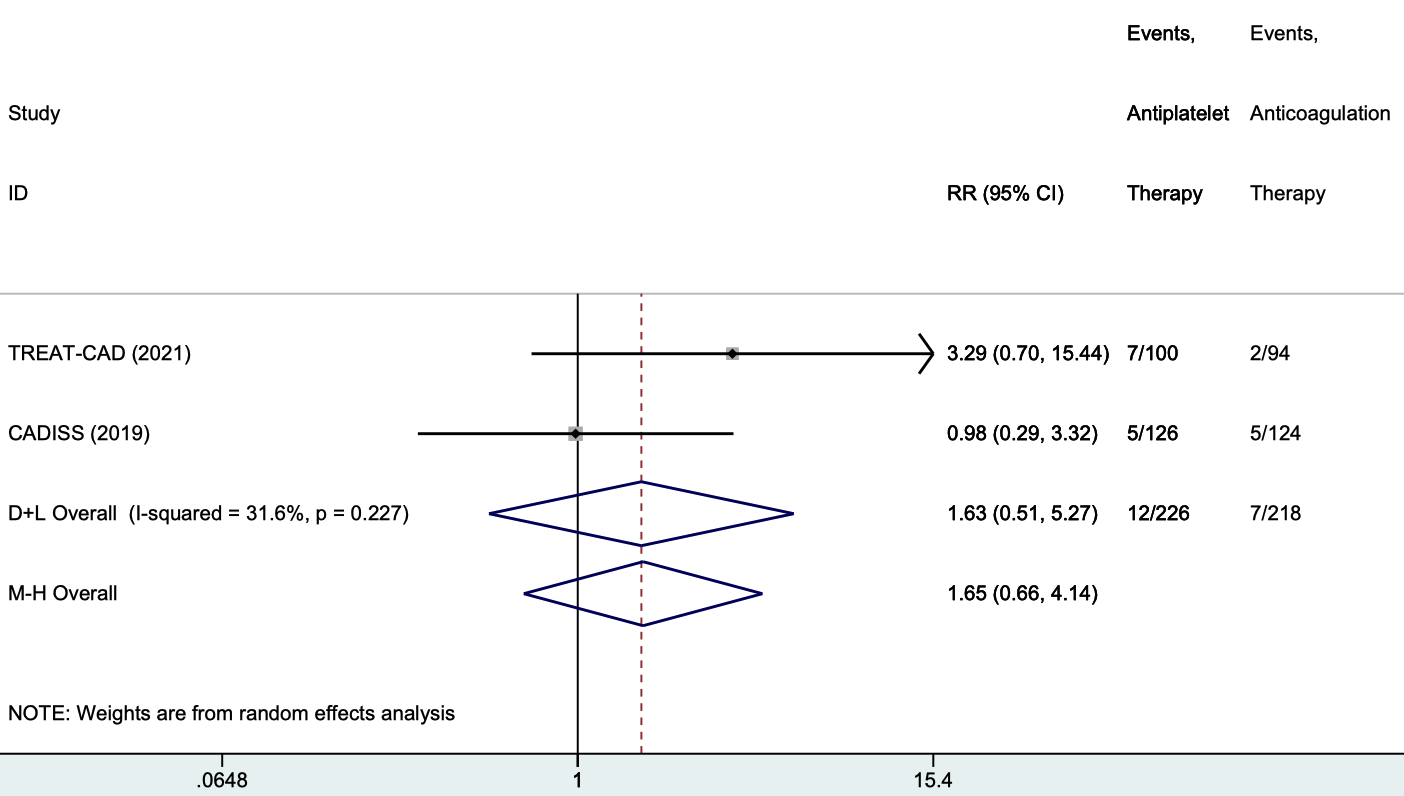
**

**Supplementary Figure 4.** Forest plot of comparison for ischemic stroke, ICH, or TIA within 3 months between antiplatelet and anticoagulation in the ITT population


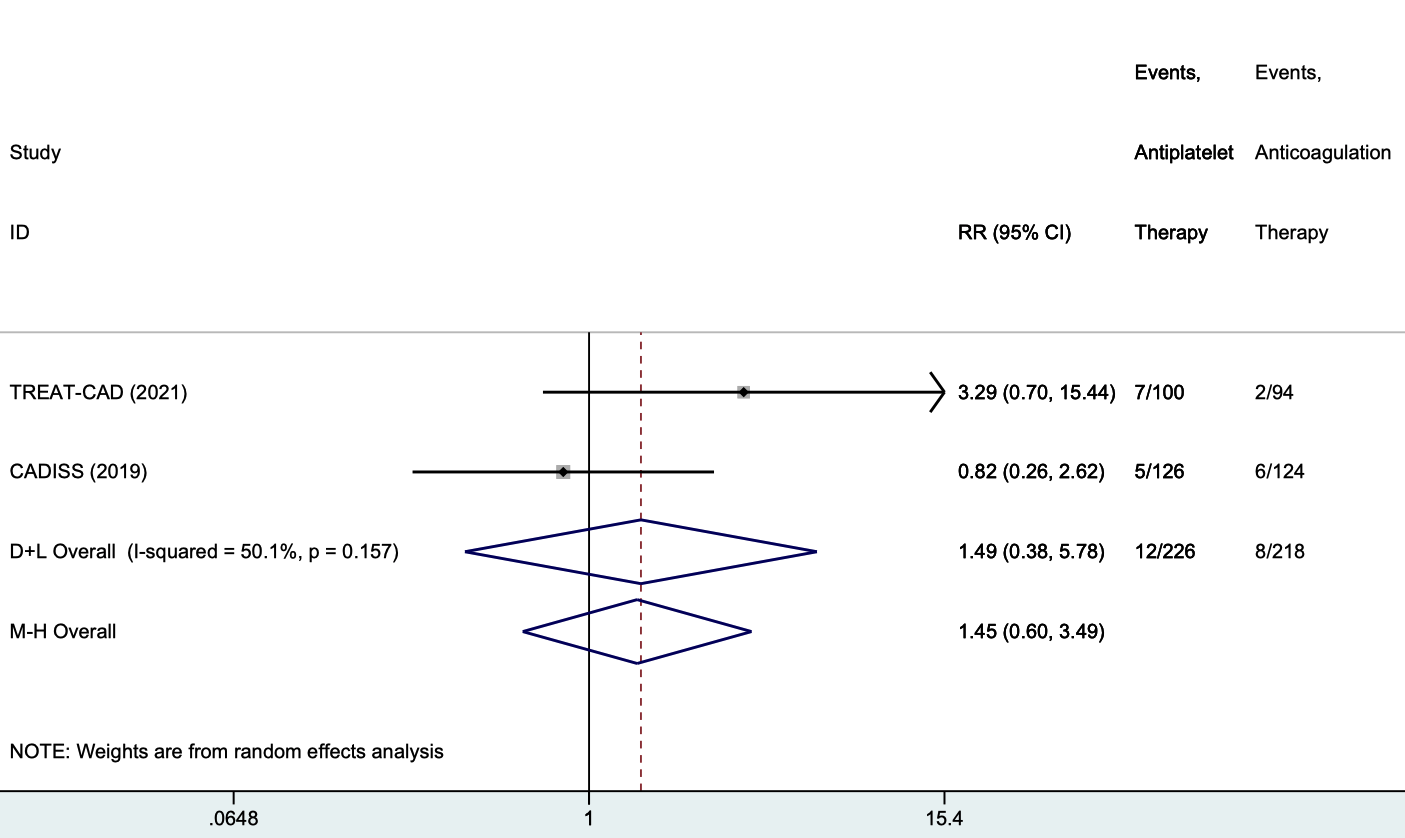


**Supplementary Figure 5.** Quality assessment of randomized controlled trials by RoB tool V.2


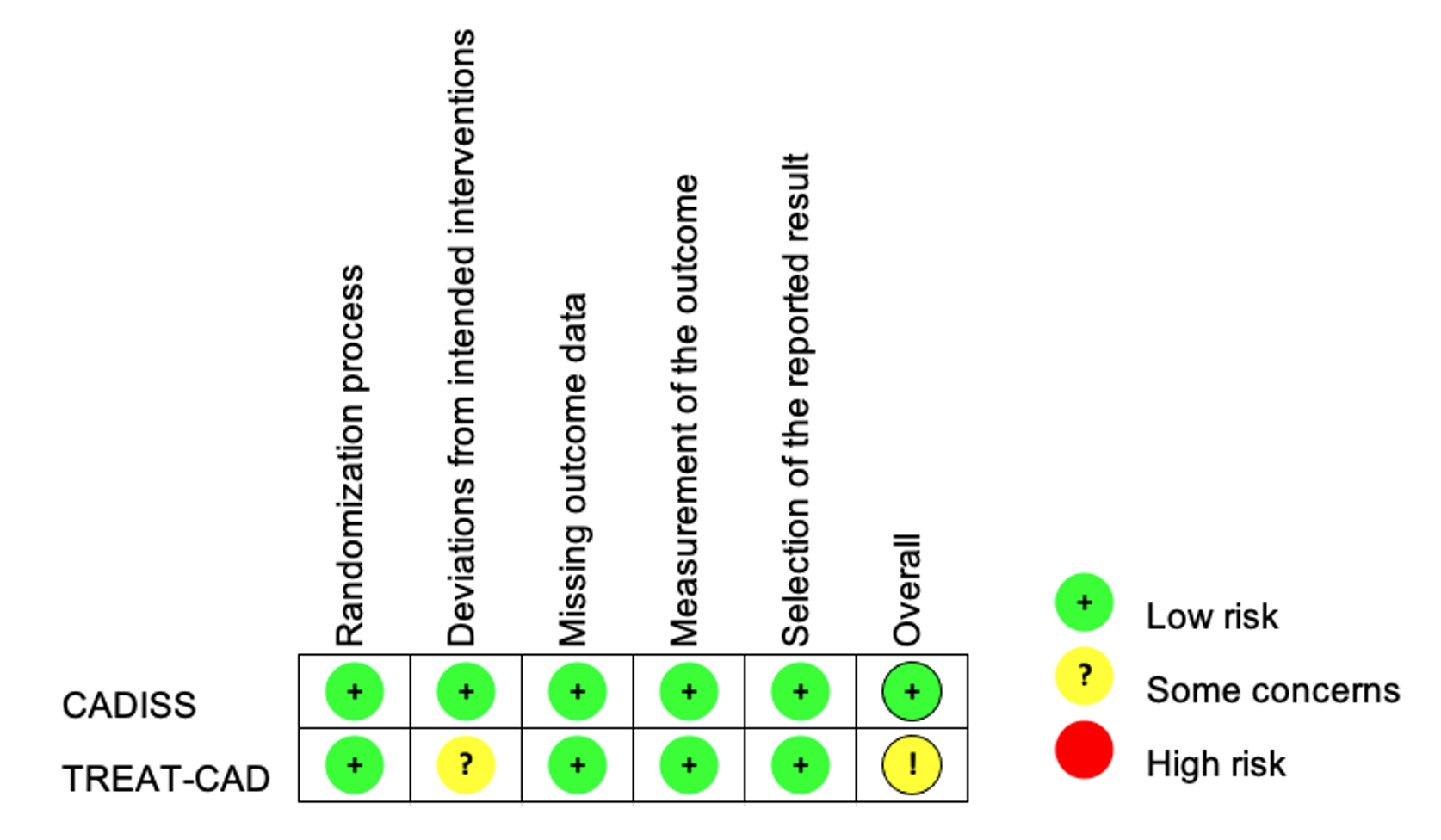

Supplement: Supplementary file 1 [file Data_Sheet_1.ZIP › Supplementary Material/Supplementary Appendix 2.docx]
