# Supplementary material for: Antiplatelet vs. Anticoagulation in Cervical Artery Dissection: A Systematic Review and Meta-Analysis of Randomized Controlled Trials
Source: Front Neurol. 2021 Nov 24;12:745106. doi: 10.3389/fneur.2021.745106 (PMC8651981; doi:10.3389/fneur.2021.745106)
Supplement: Supplementary file 1 [file Data_Sheet_1.ZIP › Supplementary Material/Supplementary Appendix 3.docx]

**Supplementary file 3.**

**Supplementary Figure 6. Funnel plots of outcomes within 3 months for antiplatelet therapy in the ITT population.**


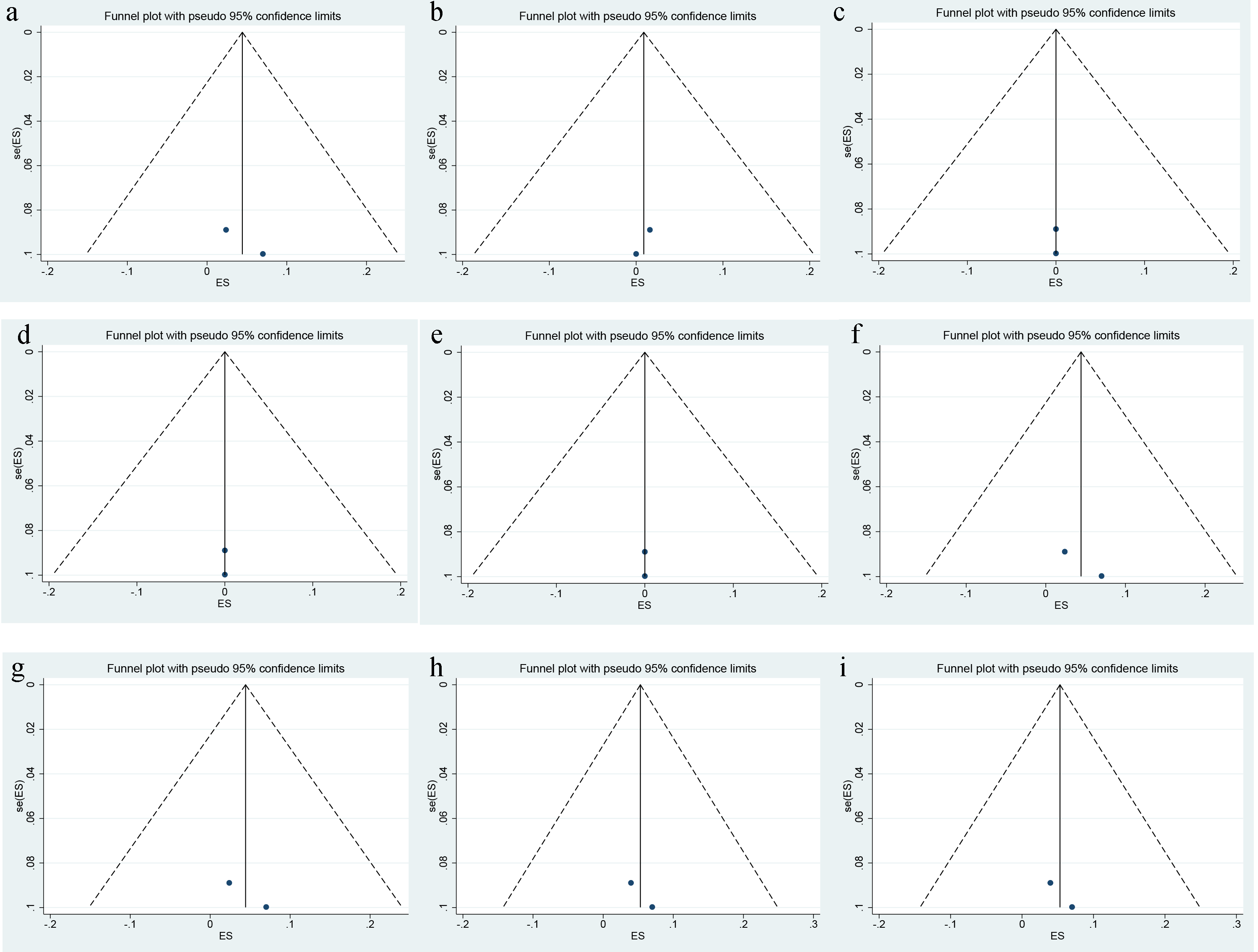


**(a)** Ischemic stroke; (**b)** TIA; (**c)** ICH; (**d)** Major extracranial bleeding; (**e)** Death; (**f)** Ischemic stroke, ICH, or death; (**g)** Ischemic stroke or ICH; (**h)** Ischemic stroke or TIA; (**i**) Ischemic stroke, ICH, or TIA.

**Supplementary Figure 7. Funnel plots of outcomes within 3 months for anticoagulation therapy in the ITT population.**


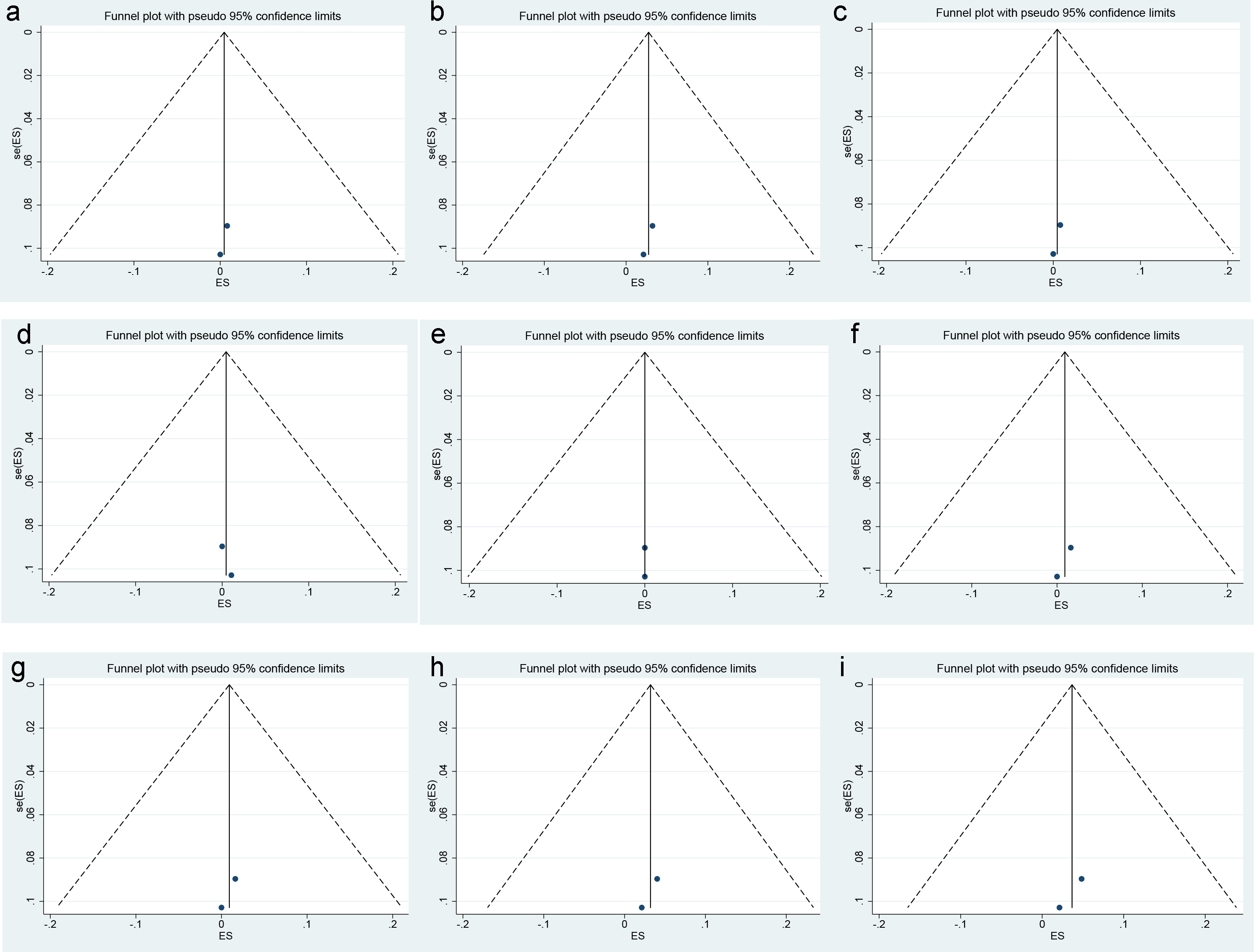


**(a)** Ischemic stroke; (**b)** TIA; (**c)** ICH; (**d)** Major extracranial bleeding; (**e)** Death; (**f)** Ischemic stroke, ICH, or death; (**g)** Ischemic stroke or ICH; (**h)** Ischemic stroke or TIA; (**i**) Ischemic stroke, ICH, or TIA.

**Supplementary Figure 8. Funnel plots of outcomes within 3 months for antiplatelet therapy in the PP population.**


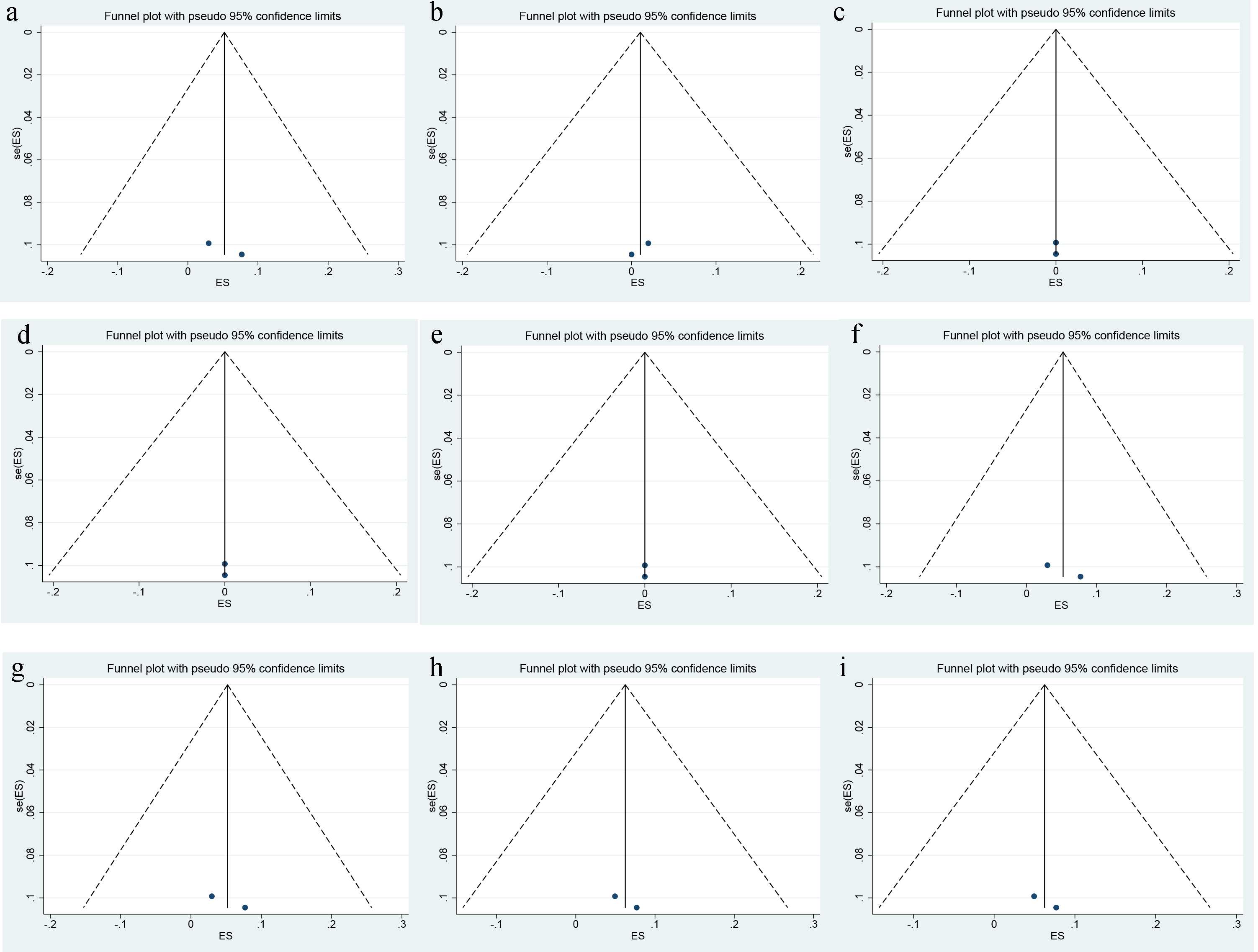


**(a)** Ischemic stroke; (**b)** TIA; (**c)** ICH; (**d)** Major extracranial bleeding; (**e)** Death; (**f)** Ischemic stroke, ICH, or death; (**g)** Ischemic stroke or ICH; (**h)** Ischemic stroke or TIA; (**i**) Ischemic stroke, ICH, or TIA.

**Supplementary Figure 9. Funnel plots of outcomes within 3 months for anticoagulation therapy in the PP population.**


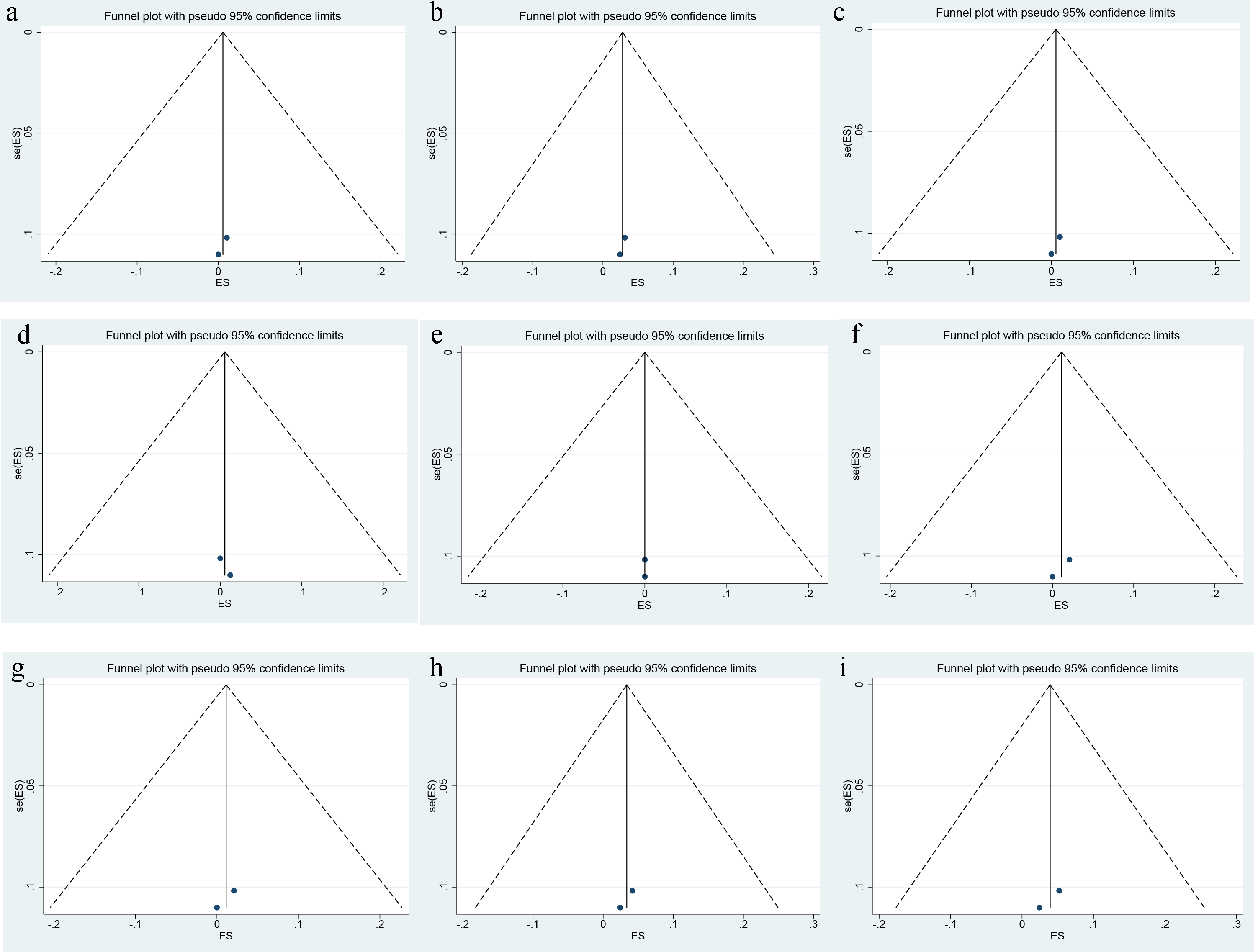


**(a)** Ischemic stroke; (**b)** TIA; (**c)** ICH; (**d)** Major extracranial bleeding; (**e)** Death; (**f)** Ischemic stroke, ICH, or death; (**g)** Ischemic stroke or ICH; (**h)** Ischemic stroke or TIA; (**i**) Ischemic stroke, ICH, or TIA.

**Supplementary Figure 10. Funnel plots of comparison for outcomes within 3 months between antiplatelet and anticoagulation therapies in the ITT population.**


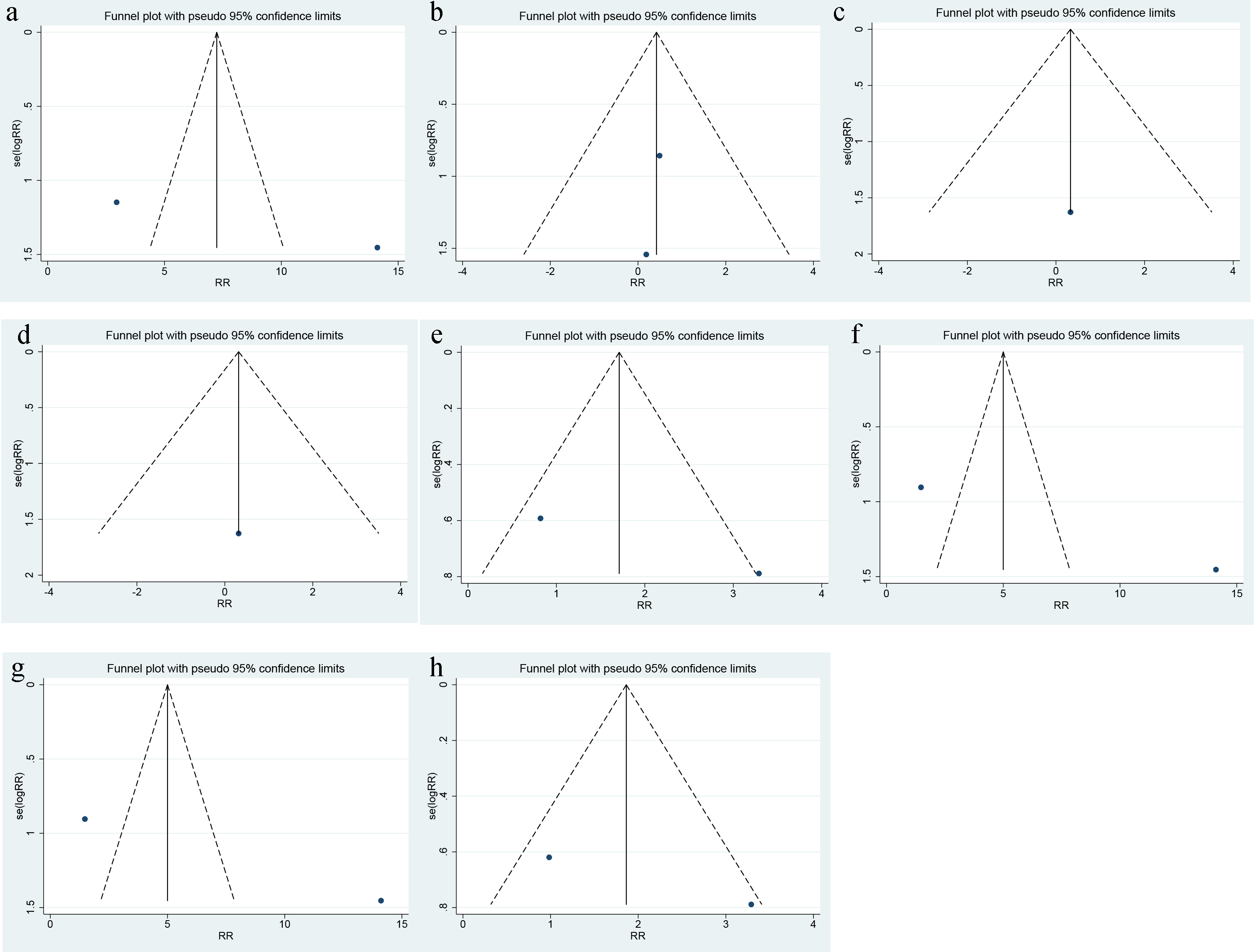


**(a)** Ischemic stroke; (**b)** TIA; (**c)** ICH; (**d)** Major extracranial bleeding; (**e)** Ischemic stroke, ICH, or TIA; (**f)** Ischemic stroke, ICH, or death; (**g)** Ischemic stroke or ICH; (**h)** Ischemic stroke or TIA.

**Supplementary Figure 11. Extfunnel plots of comparison for outcomes within 3 months between antiplatelet and anticoagulation therapies in the ITT population.**


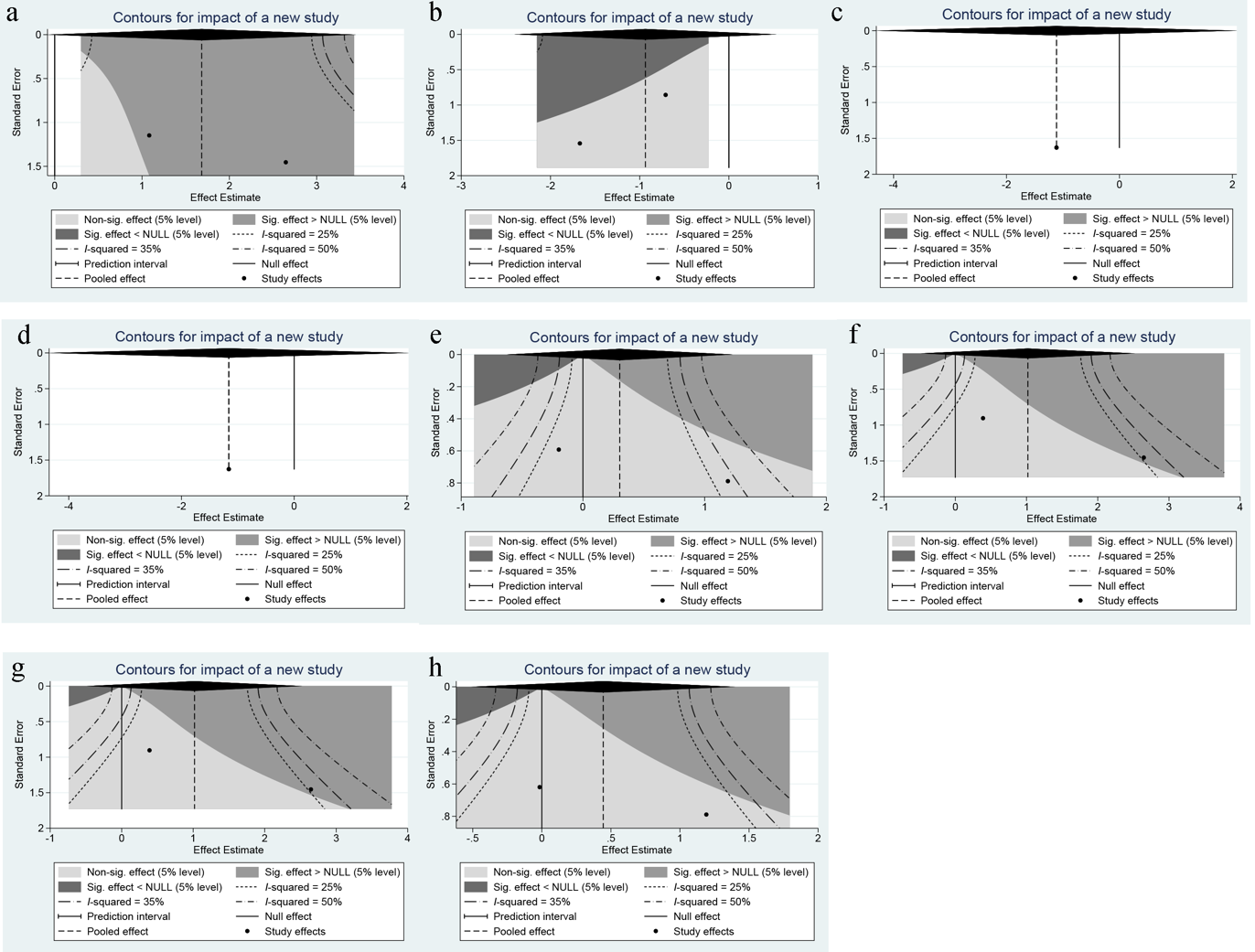


**(a)** Ischemic stroke; (**b)** TIA; (**c)** ICH; (**d)** Major extracranial bleeding; (**e)** Ischemic stroke, ICH, or TIA; (**f)** Ischemic stroke, ICH, or death; (**g)** Ischemic stroke or ICH; (**h)** Ischemic stroke or TIA.

**Supplementary Figure 12. Labbe plots of comparison for outcomes within 3 months between antiplatelet and anticoagulation therapies in the ITT population.**

**
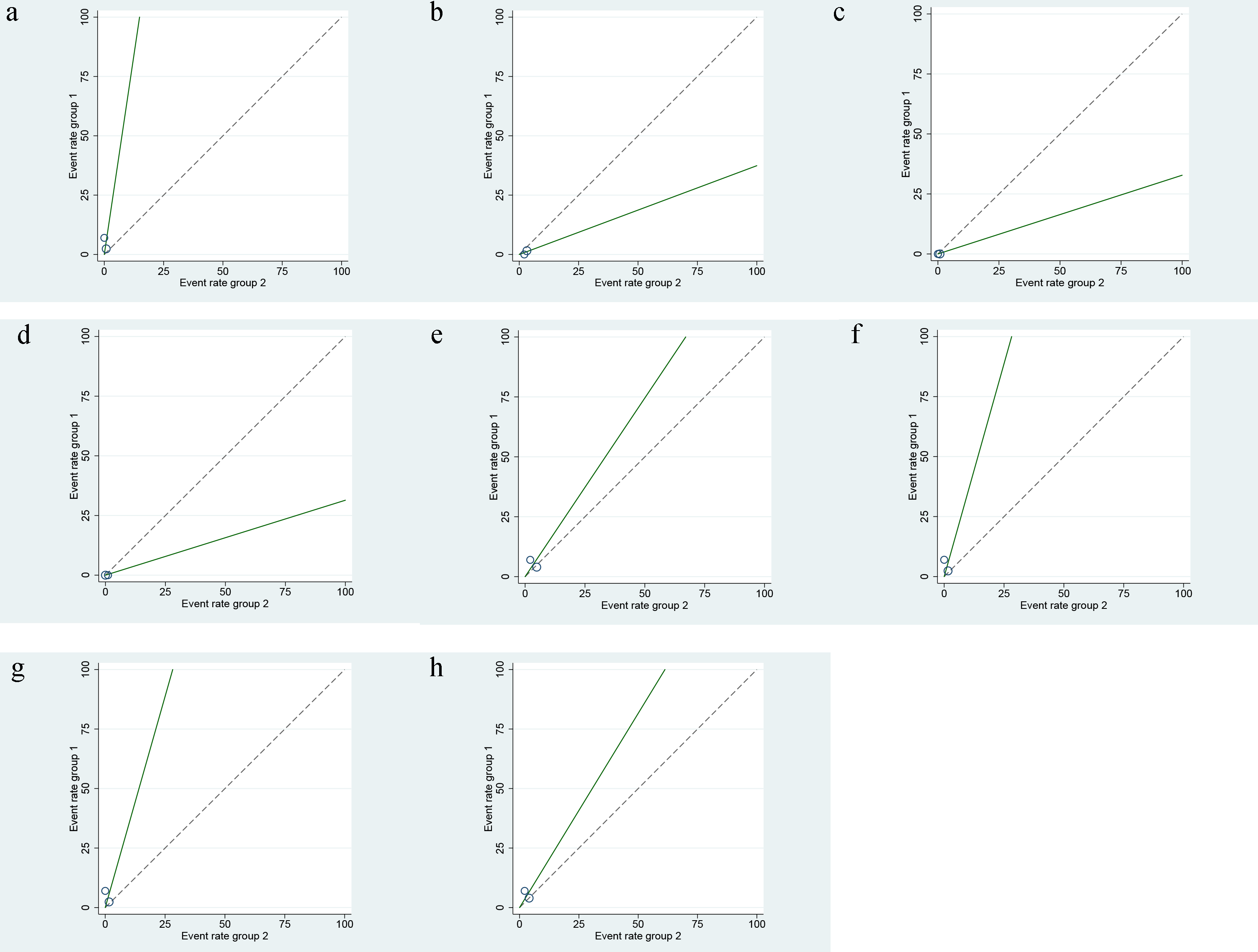
**

**(a)** Ischemic stroke; (**b)** TIA; (**c)** ICH; (**d)** Major extracranial bleeding; (**e)** Ischemic stroke, ICH, or TIA; (**f)** Ischemic stroke, ICH, or death; (**g)** Ischemic stroke or ICH; (**h)** Ischemic stroke or TIA.

**Supplementary Figure 13. Harbord tests of comparison for outcomes within 3 months between antiplatelet and anticoagulation therapies in the ITT population.**

**
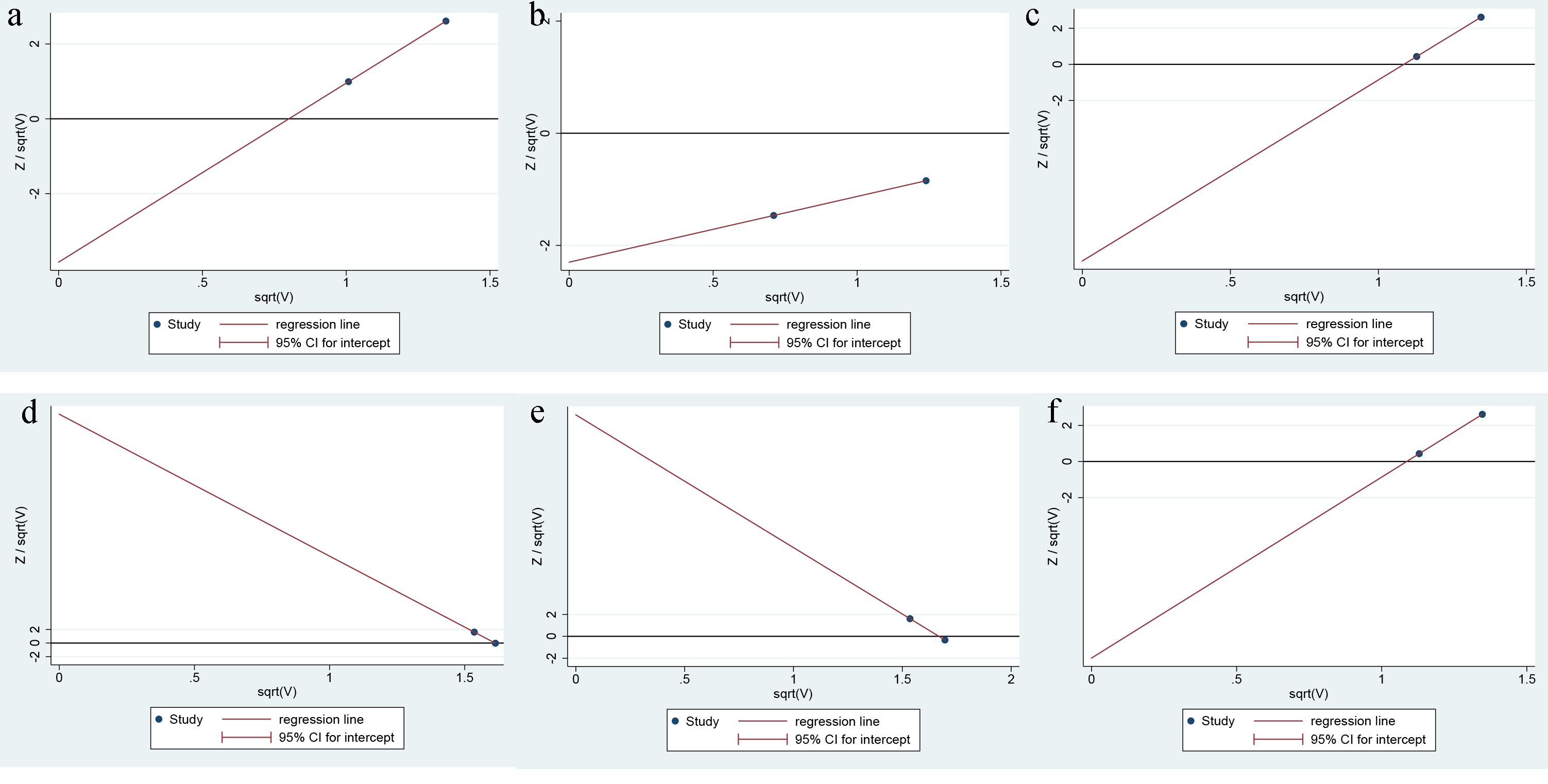
**

**(a)** Ischemic stroke; (**b)** TIA; (**c)** Ischemic stroke or ICH; (**d)** Ischemic stroke or TIA; (**e)** Ischemic stroke, ICH, or TIA; (**f)** Ischemic stroke, ICH, or death.

**Supplementary Figure 14. Sensitivity analyses of comparison for outcomes within 3 months between antiplatelet and anticoagulation therapies in the ITT population.**


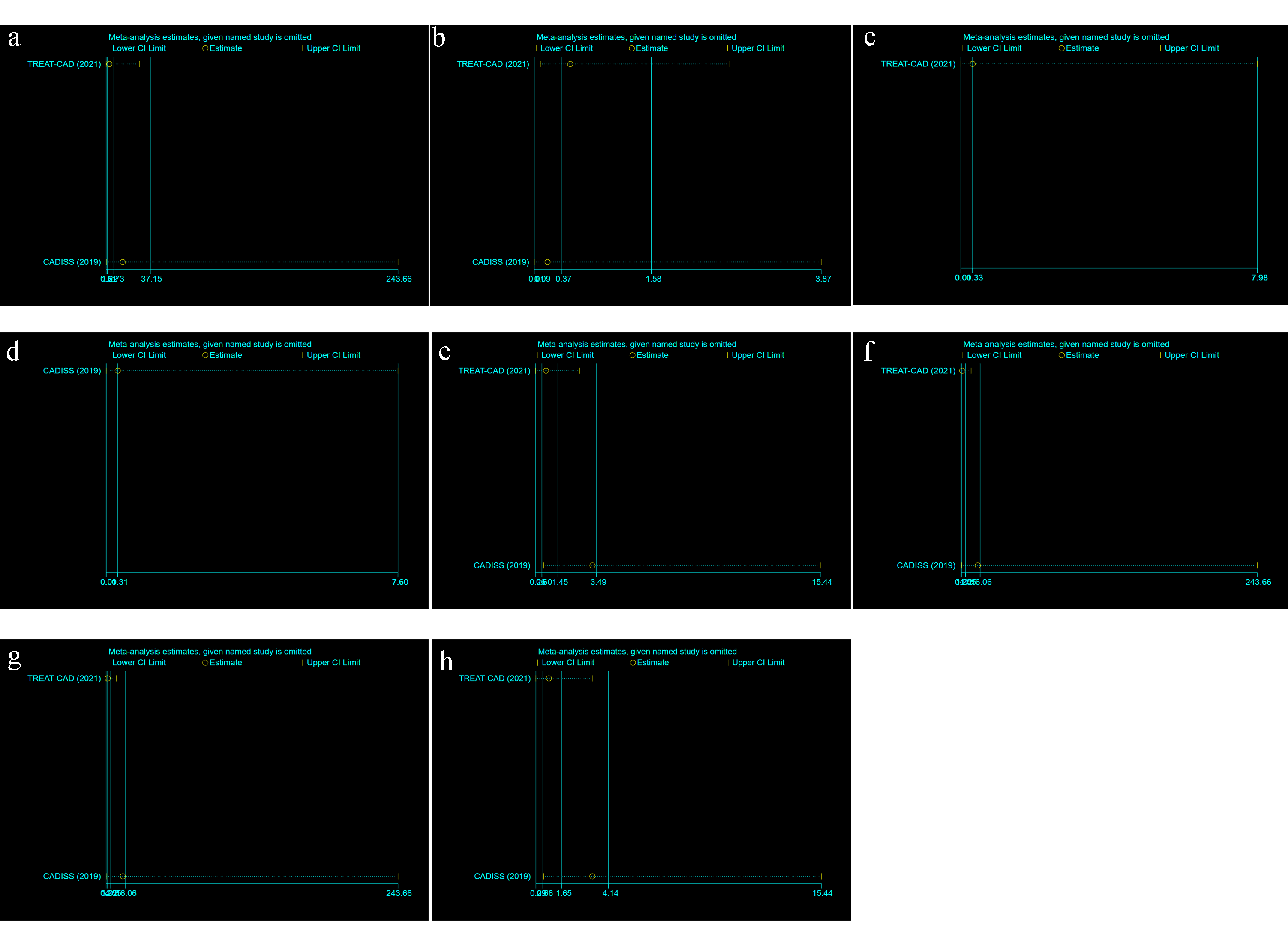


**(a)** Ischemic stroke; (**b)** TIA; (**c)** ICH; (**d)** Major extracranial bleeding; (**e)** Ischemic stroke, ICH, or TIA; (**f)** Ischemic stroke, ICH, or death; (**g)** Ischemic stroke or ICH; (**h)** Ischemic stroke or TIA.

**Supplementary Figure 15. Funnel plots of comparison for outcomes within 3 months between antiplatelet and anticoagulation therapies in the PP population.**


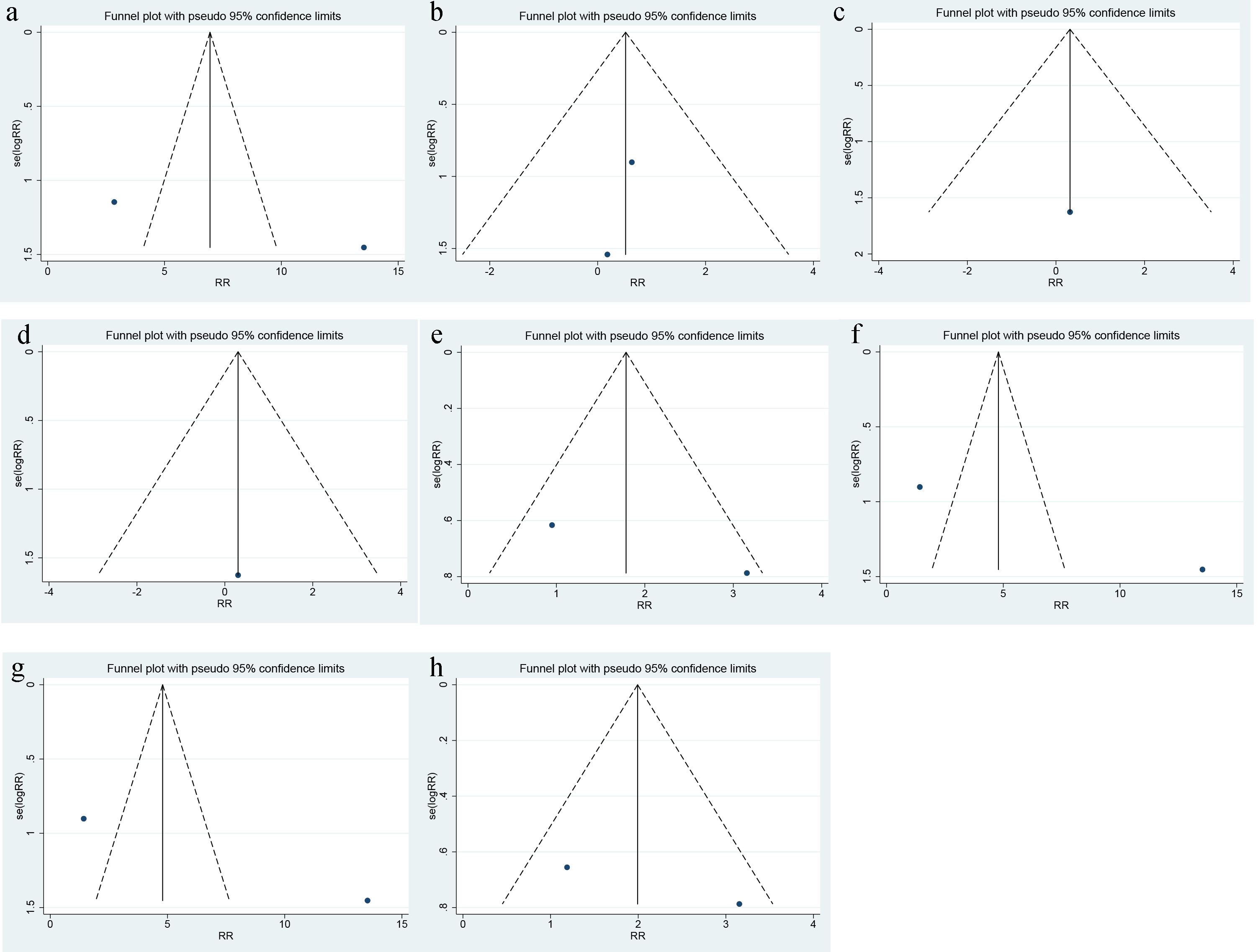


**(a)** Ischemic stroke; (**b)** TIA; (**c)** ICH; (**d)** Major extracranial bleeding; (**e)** Ischemic stroke, ICH, or TIA; (**f)** Ischemic stroke, ICH, or death; (**g)** Ischemic stroke or ICH; (**h)** Ischemic stroke or TIA.

**Supplementary Figure 16. Extfunnel plots of comparison for outcomes within 3 months between antiplatelet and anticoagulation therapies in the PP population.**


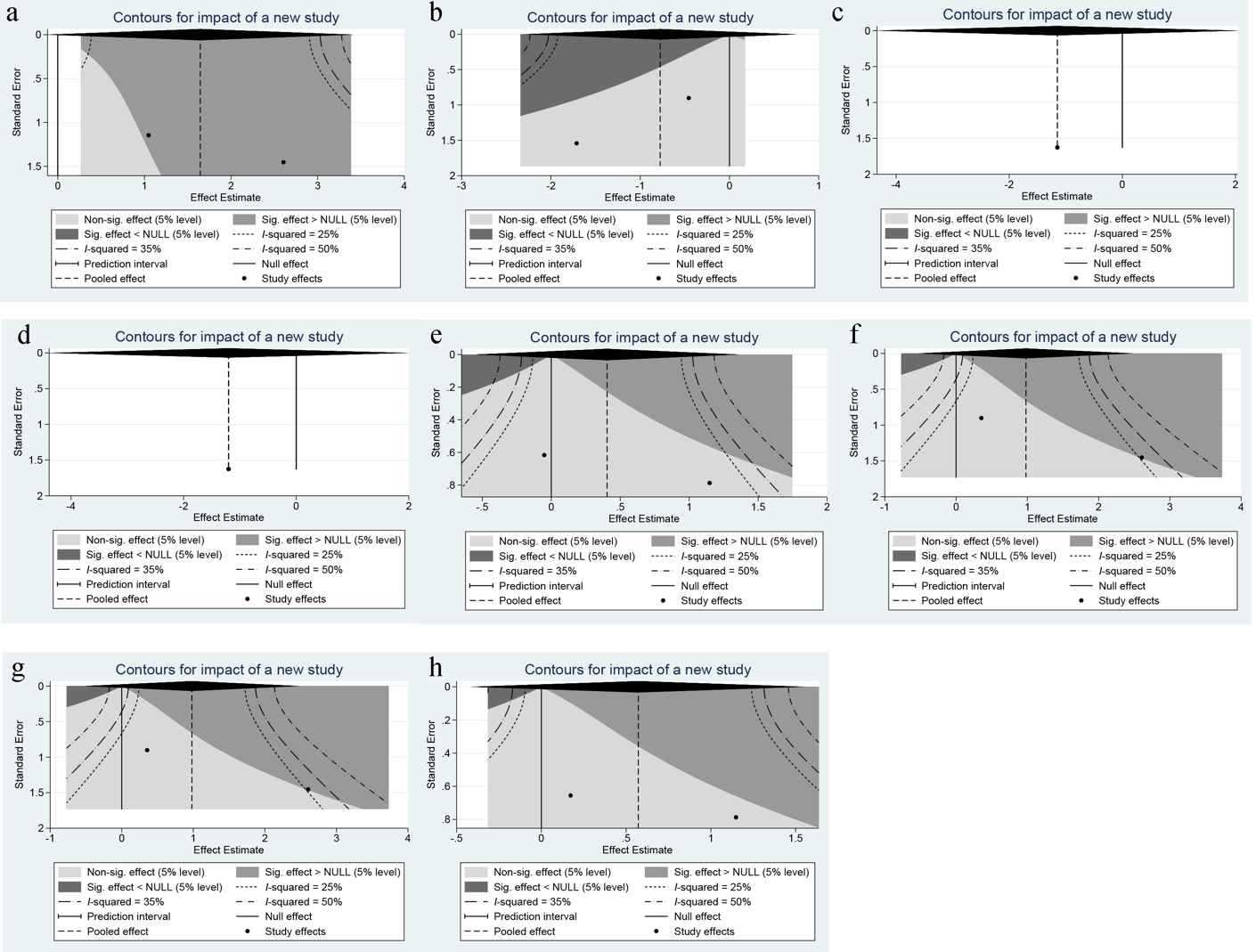


**(a)** Ischemic stroke; (**b)** TIA; (**c)** ICH; (**d)** Major extracranial bleeding; (**e)** Ischemic stroke, ICH, or TIA; (**f)** Ischemic stroke, ICH, or death; (**g)** Ischemic stroke or ICH; (**h)** Ischemic stroke or TIA.

**Supplementary Figure 17. Labbe plots of comparison for outcomes within 3 months between antiplatelet and anticoagulation therapies in the PP population.**

**
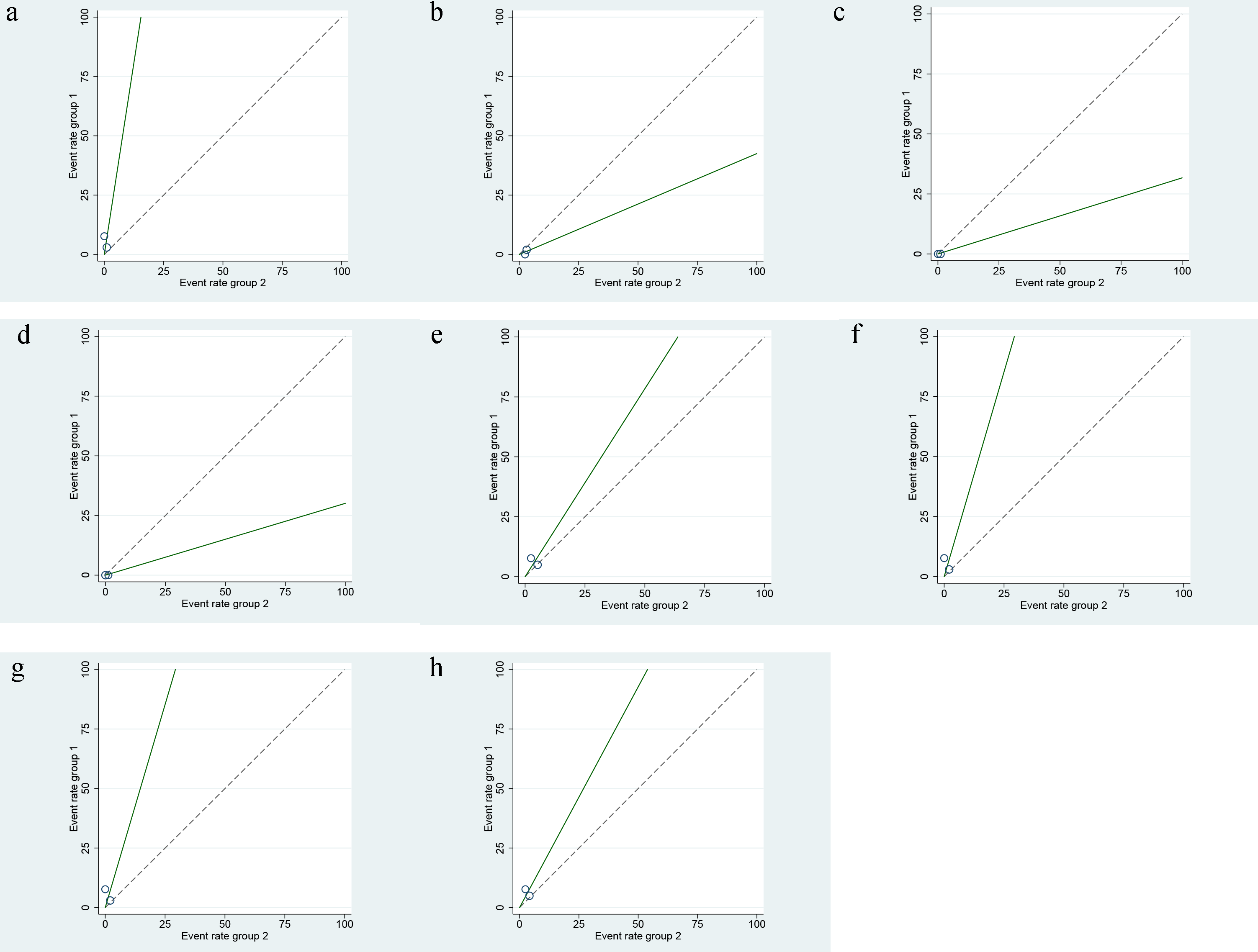
**

**(a)** Ischemic stroke; (**b)** TIA; (**c)** ICH; (**d)** Major extracranial bleeding; (**e)** Ischemic stroke, ICH, or TIA; (**f)** Ischemic stroke, ICH, or death; (**g)** Ischemic stroke or ICH; (**h)** Ischemic stroke or TIA.

**Supplementary Figure 18. Harbord tests of comparison for outcomes within 3 months between antiplatelet and anticoagulation therapies in the ITT population.**

**
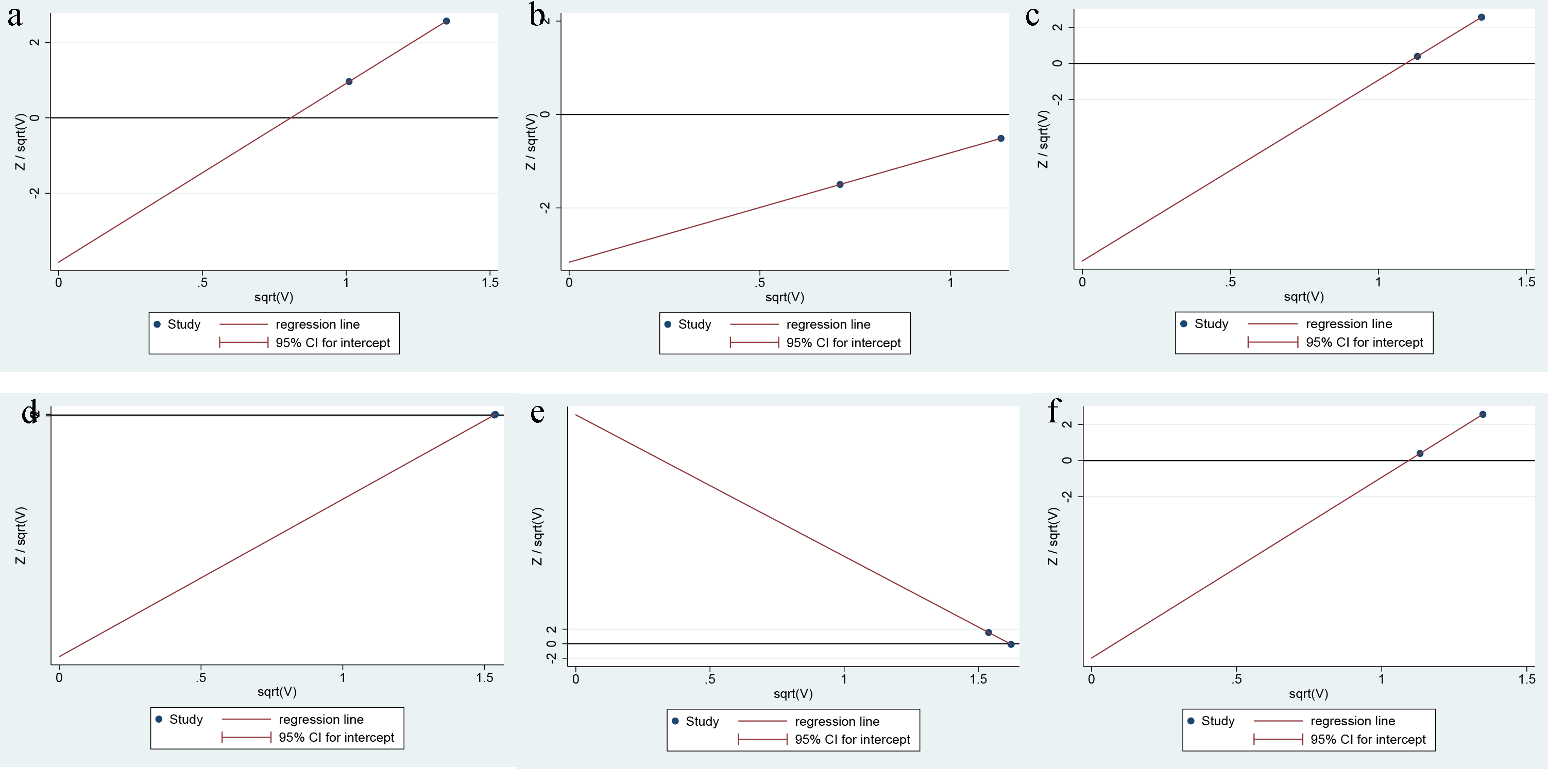
**

**(a)** Ischemic stroke; (**b)** TIA; (**c)** Ischemic stroke or ICH; (**d)** Ischemic stroke or TIA; (**e)** Ischemic stroke, ICH, or TIA; (**f)** Ischemic stroke, ICH, or death.

**Supplementary Figure 19. Sensitivity analyses of comparison for outcomes within 3 months between antiplatelet and anticoagulation therapies in the ITT population.**


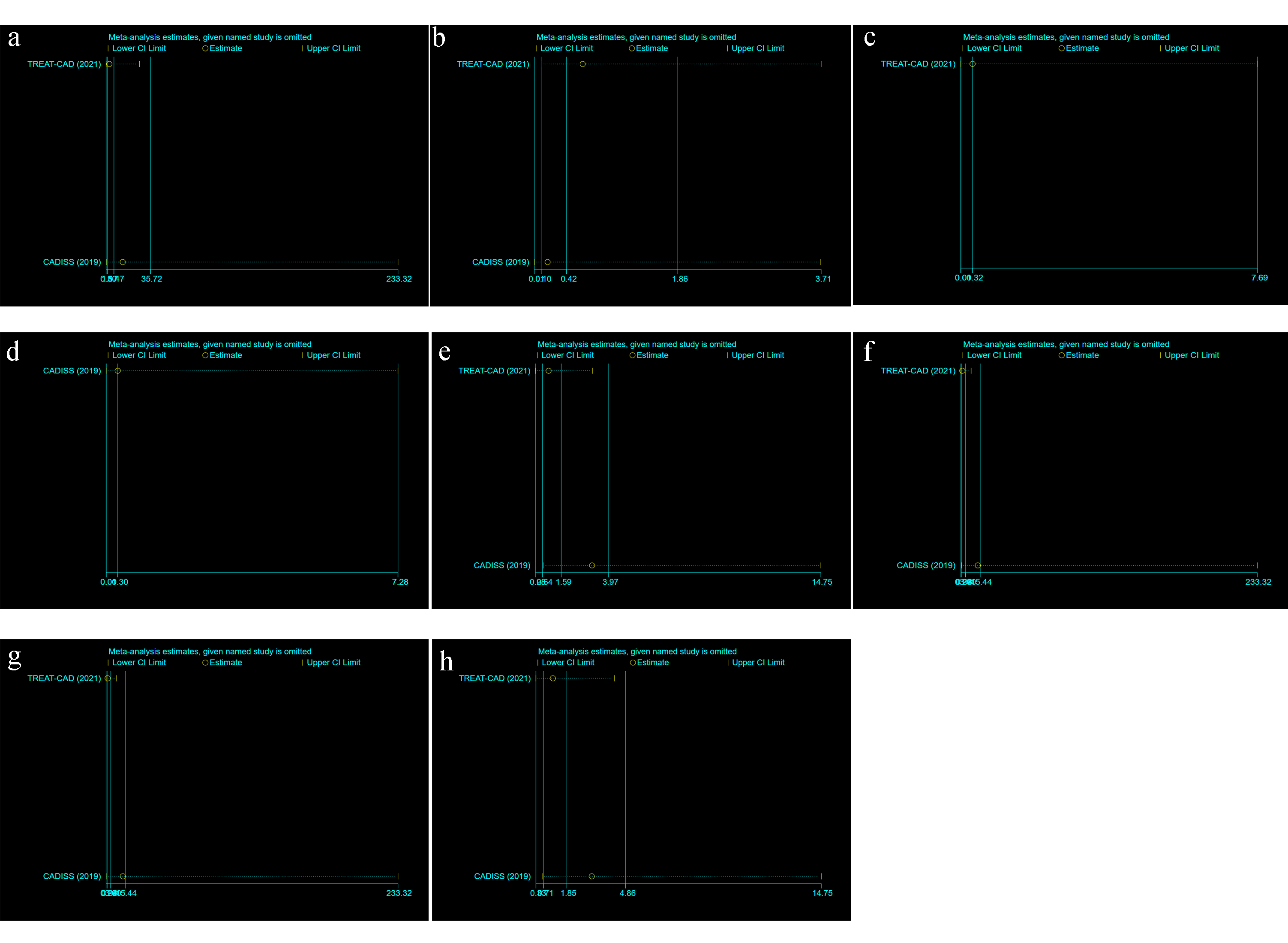


**(a)** Ischemic stroke; (**b)** TIA; (**c)** ICH; (**d)** Major extracranial bleeding; (**e)** Ischemic stroke, ICH, or TIA; (**f)** Ischemic stroke, ICH, or death; (**g)** Ischemic stroke or ICH; (**h)** Ischemic stroke or TIA.
