# Supplementary material for: Antiplatelet vs. Anticoagulation in Cervical Artery Dissection: A Systematic Review and Meta-Analysis of Randomized Controlled Trials
Source: Front Neurol. 2021 Nov 24;12:745106. doi: 10.3389/fneur.2021.745106 (PMC8651981; doi:10.3389/fneur.2021.745106)
Supplement: Supplementary file 1 [file Data_Sheet_1.ZIP › Supplementary Material/Supplementary Appendix 1.docx]

**Supplementary file 1. Search strategy**

- **MEDLINE**

**Ovid MEDLINE(R) and Epub Ahead of Print, In-Process & Other Non-Indexed Citations, Daily and Versions(R) from 1946 to May 28, 2021**

| Search step | Numbers | Searches |
| --- | --- | --- |
| 1 | 5963 | exp carotid artery injuries/ OR carotid artery, internal, dissection/ |
| 2 | 1362 | exp Vertebral Artery Dissection/ |
| 3 | 6921 | (carotid adj5 (injur* OR dissection OR trauma*)).tw. |
| 4 | 3920 | (vertebral adj5 (injur* OR dissection OR trauma*)).tw. |
| 5 | 13964 | OR/1-4 |
| 6 | 59895 | exp carotid arteries/ |
| 7 | 22500 | carotid artery diseases/ |
| 8 | 3124 | carotid artery thrombosis/ |
| 9 | 123063 | carotid*.tw. |
| 10 | 9494 | exp Vertebral Artery/ |
| 11 | 66193 | vertebral*.tw. |
| 12 | 203657 | OR/6-11 |
| 13 | 46695 | exp aneurysm, dissecting/ OR aneurysm, false/ OR exp aneurysm, ruptured/ |
| 14 | 38783 | exp wounds, nonpenetrating/ |
| 15 | 2755 | (traumatic adj5 (dissection OR aneurysm OR pseudoaneurysm)).tw. |
| 16 | 23231 | (blunt adj5 (injur* OR trauma)).tw. |
| 17 | 2552 | dissecting aneurysm.tw. |
| 18 | 45491 | rupture, spontaneous/ OR rupture/ |
| 19 | 656 | spontaneous dissection.tw. |
| 20 | 136698 | OR/13-19 |
| 21 | 8658 | 12 AND 20 |
| 22 | 18331 | 5 OR 21 |
| 23 | 127219 | exp platelet aggregation inhibitors/ |
| 24 | 10303 | blood platelets/de |
| 25 | 10182 | platelet aggregation/de |
| 26 | 174974 | exp fibrinolytic agents/ |
| 27 | 227746 | exp anticoagulants/ |
| 28 | 24613 | Thrombolytic therapy/ |
| 29 | 8024 | exp thromboembolism/dt |
| 30 | 10673 | thrombosis/dt |
| 31 | 144029 | (antiplatelet* OR antithrom* OR anticoag*).tw. |
| 32 | 57416 | (aspirin OR acetylsalicylic acid OR indobufen).tw. |
| 33 | 22746 | (dipyridamole OR ticlopidine OR clopidogrel OR sulfinpyrazone OR sulphinpyrazone).tw. |
| 34 | 122312 | (heparin* OR coumarin* OR coumadin* OR warfarin).tw. |
| 35 | 533314 | OR/23-34 |
| 36 | 2025 | 35 AND 22 |
| 37 | 144235 | randomized controlled trials as topic/ |
| 38 | 532178 | randomized controlled trial/ |
| 39 | 105381 | random allocation/ |
| 40 | 164622 | double blind method/ |
| 41 | 30254 | single blind method/ |
| 42 | 529034 | clinical trial/ |
| 43 | 21655 | clinical trial, phase i.pt |
| 44 | 34918 | clinical trial, phase ii.pt |
| 45 | 18452 | clinical trial, phase iii.pt |
| 46 | 2107 | clinical trial, phase iv.pt |
| 47 | 94182 | controlled clinical trial.pt |
| 48 | 532178 | randomized controlled trial.pt |
| 49 | 295224 | multicenter study.pt |
| 50 | 529034 | clinical trial.pt |
| 51 | 357868 | exp clinical trials as topic/ |
| 52 | 1431527 | OR/37-51 |
| 53 | 399804 | (clinical adj trial$).tw |
| 54 | 180328 | ((singl$ or doubl$ or treb$ or tripl$) adj (blind$3 or mask$3)).tw |
| 55 | 35500 | placebos/ |
| 56 | 225407 | placebo$.tw |
| 57 | 31063 | randomly allocated.tw |
| 58 | 34498 | (allocated adj2 random$).tw |
| 59 | 679918 | OR/53-58 |
| 60 | 1723173 | 52 OR 59 |
| 61 | 102 | 36 AND 60 |
| 62 | 77 | limit 61 to humans |

- **Embase**

| Search step | Numbers | Searches |
| --- | --- | --- |
| #1 | 2960 | 'carotid artery thrombosis'/dm_dt OR 'carotid artery obstruction'/dm_dt OR 'carotid artery aneurysm'/dm_dt OR 'internal carotid artery occlusion'/dm_dt OR 'internal carotid artery aneurysm'/dm_dt OR 'vertebral artery stenosis'/dm_dt |
| #2 | 160632 | 'carotid artery'/exp OR 'carotid artery disease'/exp OR 'vertebral artery'/exp |
| #3 | 257194 | carotid:ab,ti OR vertebral:ab,ti |
| #4 | 293876 | #2 OR #3 |
| #5 | 40290 | 'artery dissection'/de OR 'artery injury'/de OR 'artery rupture'/de OR 'artery thrombosis'/de |
| #6 | 1160 | 'artery'/exp AND wall AND 'dissection'/exp OR 'artery wall dissection' |
| #7 | 104511 | 'blood vessel injury'/de OR 'false aneurysm'/de OR 'blunt trauma'/de OR 'rupture'/de |
| #8 | 34837 | ('traumatic' NEAR/5 'dissection'):ab,ti OR ('traumatic' NEAR/5 'aneurysm'):ab,ti OR ('traumatic' NEAR/5 'pseudoaneurysm'):ab,ti OR ('blunt' NEAR/5 'injury'):ab,ti OR ('blunt' NEAR/5 'injuries'):ab,ti OR ('blunt' NEAR/5 'injured'):ab,ti OR ('blunt' NEAR/5 'trauma'):ab,ti OR 'dissecting aneurysm':ab,ti OR 'spontaneous dissection':ab,ti |
| #9 | 160108 | #5 OR #6 OR #7 OR #8 |
| #10 | 15780 | #4 AND #9 |
| #11 | 9289 | ('carotid' NEAR/5 'trauma'):ab,ti OR ('carotid' NEAR/5 'traumatic'):ab,ti OR ('carotid' NEAR/5 'injury'):ab,ti OR ('carotid' NEAR/5 'injuries'):ab,ti OR ('carotid' NEAR/5 'injured'):ab,ti OR ('carotid' NEAR/5 'dissection'):ab,ti |
| #12 | 3562 | ('vertebral' NEAR/5 'trauma'):ab,ti OR ('vertebral' NEAR/5 'traumatic'):ab,ti OR ('vertebral' NEAR/5 'injury'):ab,ti OR ('vertebral' NEAR/5 'injuries'):ab,ti OR ('vertebral' NEAR/5 'injured'):ab,ti |
| #13 | 26370 | #1 OR #10 OR #11 OR #12 |
| #14 | 891956 | 'anticoagulant agent'/exp OR 'antithrombocytic agent'/exp OR 'thrombocyte aggregation'/de OR 'thrombocyte'/de OR 'fibrinolytic therapy'/de OR 'thromboembolism'/de |
| #15 | 173766 | antiplatelet*:ab,ti OR anticoagulant*:ab,ti OR antithromb*:ab,ti |
| #16 | 88628 | aspirin:ab,ti OR 'acetylsalicylic acid':ab,ti OR indobufen:ab,ti |
| #17 | 37120 | dipyridamole:ab,ti OR ticlopidine:ab,ti OR clopidogrel:ab,ti OR sulfinpyrazone:ab,ti OR sulphinpyrazone:ab,ti |
| #18 | 174338 | heparin*:ab,ti OR coumarin*:ab,ti OR coumadin*:ab,ti OR warfarin:ab,ti |
| #19 | 971251 | #14 OR #15 OR #16 OR #17 OR #18 |
| #20 | 6887 | #13 AND #19 |
| #21 | 1826186 | 'clinical trial'/de OR 'randomized controlled trial'/de OR 'controlled clinical trial'/de OR 'multicenter study'/de OR 'phase 3 clinical trial'/de OR 'phase 4 clinical trial'/de OR 'randomization'/exp OR 'single blind procedure'/de OR 'double blind procedure'/de OR 'crossover procedure'/de OR 'placebo'/de |
| #22 | 1230929 | (randomi?ed NEAR/2 controlled NEAR/2 trial*) OR rct OR placebo* |
| #23 | 46672 | random* NEAR/2 allocat* |
| #24 | 323802 | (single OR double OR treble OR triple) NEAR/2 blind* |
| #25 | 2131630 | #21 OR #22 OR #23 OR #24 |
| #26 | 754 | #20 AND #25 |
| #27 | 697 | #20 AND #25 AND [humans]/lim |

- **Cochrane Central Register of Controlled Trials (CENTRAL)**

| **Search step** | **Numbers** | **Searches** |
| --- | --- | --- |
| #1 | 15 | MeSH descriptor: [Carotid Artery Injuries] this term only |
| #2 | 6 | MeSH descriptor: [Carotid Artery, Internal, Dissection] this term only |
| #3 | 8 | MeSH descriptor: [Vertebral Artery Dissection] this term only |
| #4 | 66 | MeSH descriptor: [Vertebral Artery] this term only |
| #5 | 249 | MeSH descriptor: [Neck Injuries] explode all trees |
| #6 | 255 | (((carotid or vertebr* or cervical) near/2 arter* near/3 (dissect* or damag* or injur* or lesion* or laceration* or trauma* or ruptur* or wound))):ti,ab,kw (Word variations have been searched) |
| #7 | 563 | #1 or #2 or #3 or #4 or #5 or #6 |
| #8 | 1137 | MeSH descriptor: [Carotid Arteries] explode all trees |
| #9 | 486 | MeSH descriptor: [Carotid Artery Diseases] this term only |
| #10 | 18 | MeSH descriptor: [Carotid Artery Thrombosis] this term only |
| #11 | 1433 | #8 or #9 or #10 |
| #12 | 105 | MeSH descriptor: [Aneurysm, Dissecting] this term only |
| #13 | 27 | MeSH descriptor: [Aneurysm, False] this term only |
| #14 | 131 | MeSH descriptor: [Aneurysm, Ruptured] this term only |
| #15 | 758 | MeSH descriptor: [Wounds, Nonpenetrating] explode all trees |
| #16 | 110 | MeSH descriptor: [Rupture, Spontaneous] this term only |
| #17 | 16 | ((blunt near/5 (injur* or trauma))):ti,ab,kw (Word variations have been searched) |
| #18 | 530 | (dissecting aneurysm):ti,ab,kw (Word variations have been searched) |
| #19 | 142 | ((traumatic near/5 (dissection or aneurysm or pseudoaneurysm))):ti,ab,kw (Word variations have been searched) |
| #20 | 1583 | #12 or #13 or #14 or #15 or #16 or #17 or #18 or #19 |
| #21 | 8 | #11 and #20 |
| #22 | 569 | #7 or #21 |
| #23 | 4741 | MeSH descriptor: [Anticoagulants] explode all trees |
| #24 | 12670 | (anticoagulant* or anti‐coagulant* or apixaban or dabigatran or endosaban or rivaroxaban):ti,ab,kw |
| #25 | 924 | MeSH descriptor: [Antithrombins] explode all trees |
| #26 | 5446 | (antithromb* or anti‐thromb*):ti,ab,kw |
| #27 | 2302 | MeSH descriptor: [Coumarins] explode all trees |
| #28 | 413 | (coumarin* or chromonar or coumestrol or esculin or isocoumarin* or psoralens or pyranocoumarins or umbelliferones):ti,ab,kw |
| #29 | 1723 | MeSH descriptor: [Warfarin] explode all trees |
| #30 | 5116 | (warfarin or coumadine or warfant or coumadin or marevan or aldocumar or tedicumar):ti,ab,kw |
| #31 | 4016 | MeSH descriptor: [Platelet Aggregation Inhibitors] explode all trees |
| #32 | 11574 | (alprostadil or dipyridamol* or disintegrin or epoprostenol or iloprost or ketanserin or milrinone or pentoxifylline or s‐nitrosoglutathione or nitrosothiols or ticlopidine or clopidogrel or trapidil):ti,ab,kw |
| #33 | 5095 | antiplatelet near/1 (therap* or regime* or treatment* or intervention* or agent* or drug*):ti,ab,kw |
| #34 | 378 | platelet near/1 (antiaggreg* or anti‐aggreg* or inhibitor* or antagonist*):ti,ab,kw |
| #35 | 18568 | (aspirin* or acetylsalicylic acid or salicylat* or salicylic*):ti,ab,kw |
| #36 | 42771 | #23 or #24 or #25 or #26 or #27 or #28 or #29 or #30 or #31 or #32 or #33 or #34 or #35 |
| #37 | 49 | #22 and #36 |
